# Linear Conjugated Polymers for Solar-Driven Hydrogen Peroxide Production: The Importance of Catalyst Stability

**DOI:** 10.1021/jacs.1c09979

**Published:** 2021-11-10

**Authors:** Lunjie Liu, Mei-Yan Gao, Haofan Yang, Xiaoyan Wang, Xiaobo Li, Andrew I. Cooper

**Affiliations:** † Department of Chemistry and Materials Innovation Factory, University of Liverpool, 51 Oxford Street, Liverpool L7 3NY, United Kingdom; ‡ Department of Chemical Sciences, Bernal Institute, University of Limerick, Limerick V94 T9PX, Republic of Ireland

## Abstract

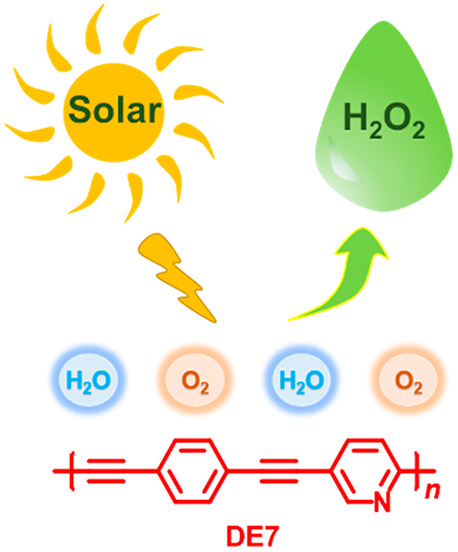

Hydrogen peroxide (H_2_O_2_) is one of the most important industrial oxidants. In principle, photocatalytic H_2_O_2_ synthesis from oxygen and H_2_O using sunlight could provide a cleaner alternative route to the current anthraquinone process. Recently, conjugated organic materials have been studied as photocatalysts for solar fuels synthesis because they offer synthetic tunability over a large chemical space. Here, we used high-throughput experiments to discover a linear conjugated polymer, poly(3-4-ethynylphenyl)ethynyl)pyridine (DE7), which exhibits efficient photocatalytic H_2_O_2_ production from H_2_O and O_2_ under visible light illumination for periods of up to 10 h or so. The apparent quantum yield was 8.7% at 420 nm. Mechanistic investigations showed that the H_2_O_2_ was produced via the photoinduced stepwise reduction of O_2_. At longer photolysis times, however, this catalyst decomposed, suggesting a need to focus the photostability of organic photocatalysts, as well as the initial catalytic production rates.

Hydrogen peroxide (H_2_O_2_) is used on a huge scale in applications, such as paper and textile bleaching, chemical synthesis, wastewater treatment, and (on a smaller scale) fuel cells.^1^ H_2_O_2_ is mainly prepared via the anthraquinone process, which uses large amounts of energy and creates a lot of chemical waste.^2^ The clean production of H_2_O_2_ via the photocatalytic reaction of H_2_O and O_2_ under solar illumination has therefore sparked much recent interest.^3^

To achieve efficient H_2_O_2_ production, photocatalysts must absorb sunlight, produce separated charges, and drive redox reactions. A variety of organic materials have been investigated, including graphitic carbon nitride (g-C_3_N_4_),^4−7^ supramolecular coordination complexes,^8^ covalent organic frameworks (COFs),^9^ triazine-based frameworks (CTFs),^10^ and polymer resins.^11−13^ Recently, promising H_2_O_2_ production efficiencies were reported for some organic photocatalysts.^5,11−13^ However, to date, few organic photocatalysts have shown good performance for photocatalytic H_2_O_2_ synthesis without sacrificial agents, and efficiencies are far from satisfying industrial requirements.

Here we used high-throughput experiments to identify a linear conjugated polymer (LCP), poly(3-4-ethynylphenyl)ethynyl)pyridine (DE7), with good activity for photocatalytic H_2_O_2_ production from H_2_O and O_2_ under visible light. The apparent quantum yield (AQY) of DE7 was 8.7% at 420 nm over short irradiation times, but over longer periods (>50 h), both DE7 and a resorcinol-formaldehyde catalyst^11−13^ were found to become deactivated.

An initial library of 60 candidate materials was designed (Figure 1a–f and Figures S1–4) that included g-C_3_N_4_, TiO_2_, and known photocatalytic conjugated polymers (P1, P7, and P10)^14^ as benchmarks. This functionally diverse library of branched and linear conjugated polymers was then screened for H_2_O_2_ production in pure water (no added sacrificial donors) in air using a modified high-throughput screening platform.^15^ DE7 showed the highest H_2_O_2_ production in this library over an irradiation period of 1.5 h using simulated solar light (Figure 1a). Other derivatives of DE7 were also synthesized (DE7-D-n, Figures S5–S7), but DE7 still showed the highest activity. We found no simple correlation between the H_2_O_2_ production rate and any single physical property such as band gap, fluorescence lifetime, particle size, or water contact angle (Figure S8), much as for sacrificial hydrogen evolution.^15,16^

**Figure 1:**
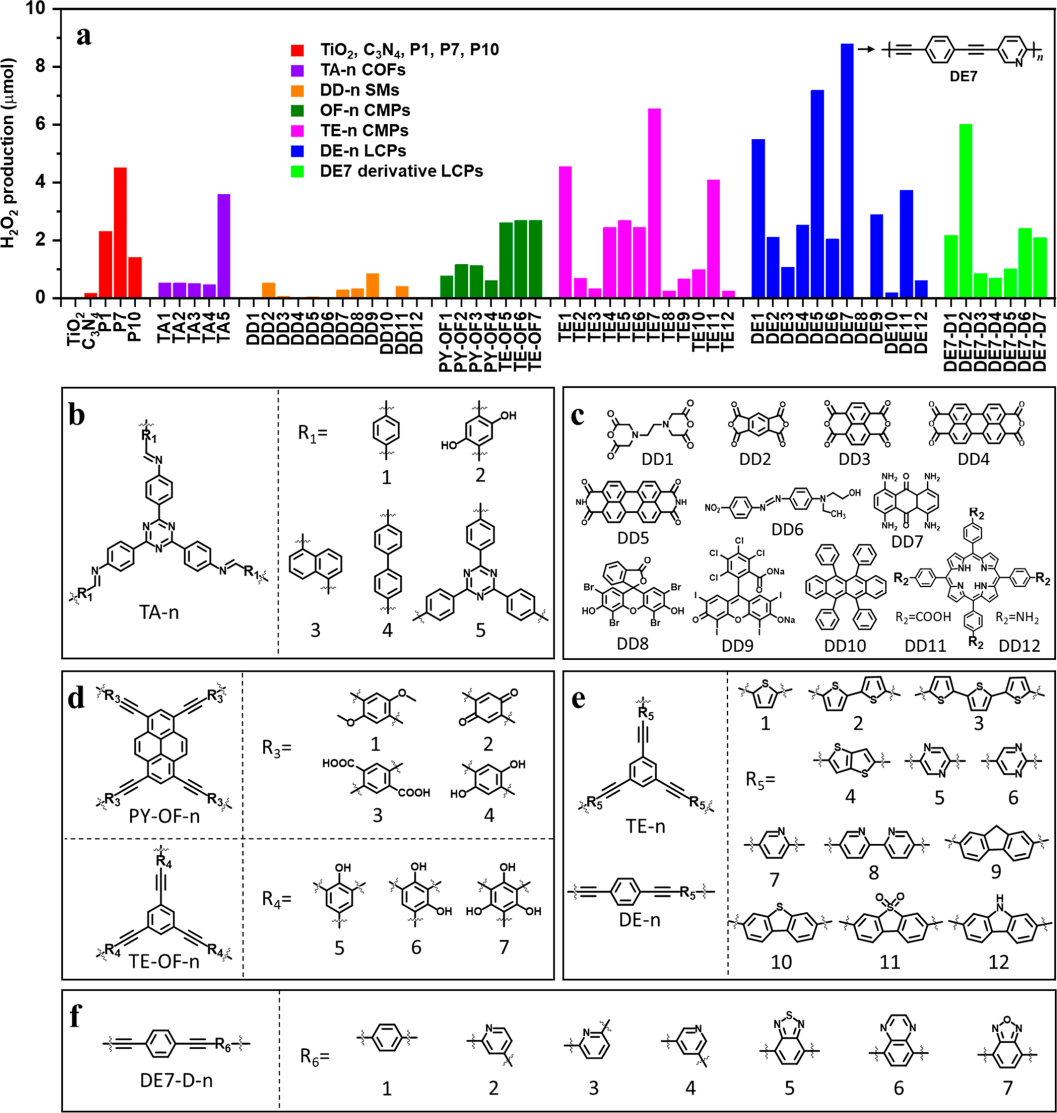
(a) High-throughput discovery of organic materials for photocatalytic H_2_O_2_ production. Reaction conditions: 5 mg of polymer, 3 mL of H_2_O, air, simulated solar light, 1.5 h. Structures of TA-n COFs (b), small molecules, DD-n (c), PY/TE-OF-n CMPs (d), TE-n CMPs and DE-n LCPs (e), and DE7 LCP analogues (f).

DE7 shows poor activity for photocatalytic hydrogen (H_2_) evolution,^17^ and more broadly, sacrificial H_2_ production and H_2_O_2_ generation are somewhat contraindicated (Figure S9), although several catalysts (e*.*g., DE3, TE2, TE3, TE9, TE10, TE12) are poor catalysts for both reactions. DE7 was synthesized by Pd-catalyzed Sonogashira coupling of 1,4-diethynylbenzene and 2,5-dibromopyridine (Figures S10–S15). To investigate the effect of residual metals^18,19^ on catalytic performance, DE7 was prepared with different amounts of Pd(PPh_3_)Cl_2_ and CuI. Excessive quantities of Pd and Cu in the polymer decreased the catalytic performance, although only at very high metal loadings (Figure S16), perhaps due to Fenton-like reactions^20^ or effects on charge transfer pathways.^19^ Other Sonogashira-coupled polymers contained variable quantities of Cu (Table S1; mostly <0.5 wt %). Polymers in the DE7-n series had similar Cu contents to DE7 but showed significantly lower catalytic performance. As such, we do not believe that variable Cu contents in these polymers account for their variable catalytic performances. Microwave heating was also used to synthesize DE7-M (that is, DE7 synthesized under microwave conditions), shortening the synthesis time from 2 days^17^ to 2 h along with enhanced photoelectric properties (Figures S17 and S18). The optical gap of DE7-M was calculated from the UV–visible spectrum to be 2.34 eV (Figure 2a). Powder X-ray diffraction (PXRD) patterns (Figure 2b) showed that DE7-M was semicrystalline. The polymer mainly consisted of plicated sheets (Figure 2c,d). The contact angle of DE7-M against water was 67°, indicating moderate wetting behavior (Figure S19).

**Figure 2:**
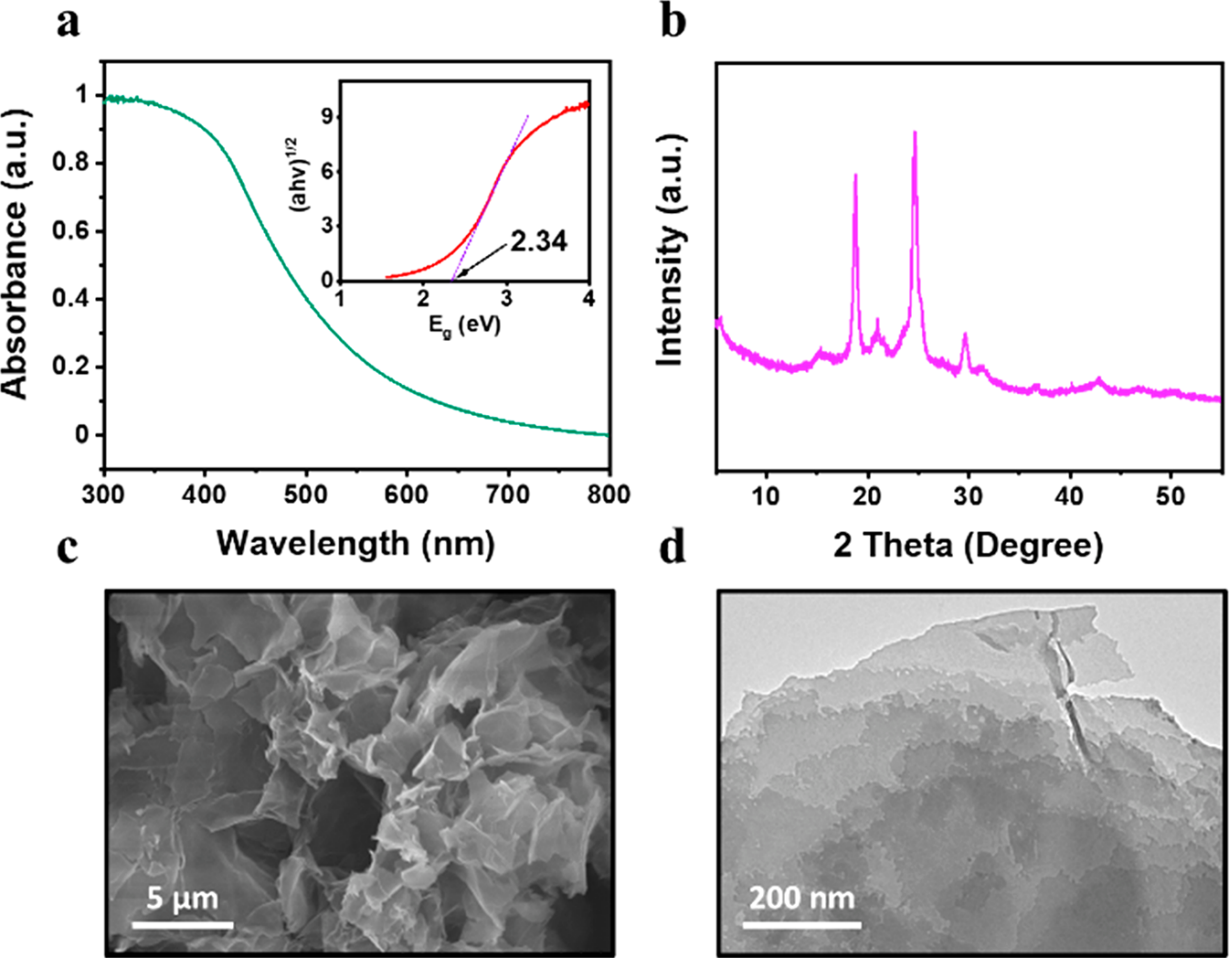
(a) Solid-state UV–visible spectrum for DE7-M; inset figure shows a Tauc plot. (b) PXRD pattern, (c) SEM, and (d) TEM image for DE7-M.

The AQY was measured at different wavelengths to evaluate the photocatalytic H_2_O_2_ production performance. The AQY was determined to be 8.7% at 420 nm (Figure 3a), which is higher than most reported organic photocatalysts for H_2_O_2_ production using pure water, such as PEI/C_3_N_4_ (2.21% at 420 nm)^21^ and RF523 (∼8.0% at 420 nm),^11^ but lower than Sb-SAPC15 (17.6% at 420 nm)^5^ and OCN-500 (10.2% at 420 nm)^4^ (Table S2). The AQY profile followed the absorption spectrum, supporting a photoinduced H_2_O_2_ generation process (Figure 3a). The solar-to-chemical conversion efficiency was 0.28% in the first hour, falling to 0.23% after 5 h (Figure S20). As shown in Figure 3b, no H_2_O_2_ was detected under a nitrogen atmosphere. The amount of H_2_O_2_ produced under pure O_2_ (99%) was 1.6 times greater than in air, again indicating that O_2_ is essential for H_2_O_2_ production. This was further confirmed by isotopic labeling experiments using ^18^O_2_ (Figure 3c): the percentage of ^18^O_2_ detected by mass spectroscopy increased from 0% to 59% over 24 h. Electron paramagnetic resonance (EPR) measurements detected radicals using 5,5-dimethyl-1-pyrroline *N*-oxide (DMPO) as the trapping agent (Figure 3d). The typical signals of **^•^**O_2_^–^ were found after illumination,^21^ indicating the one-electron reduction of O_2_.

**Figure 3:**
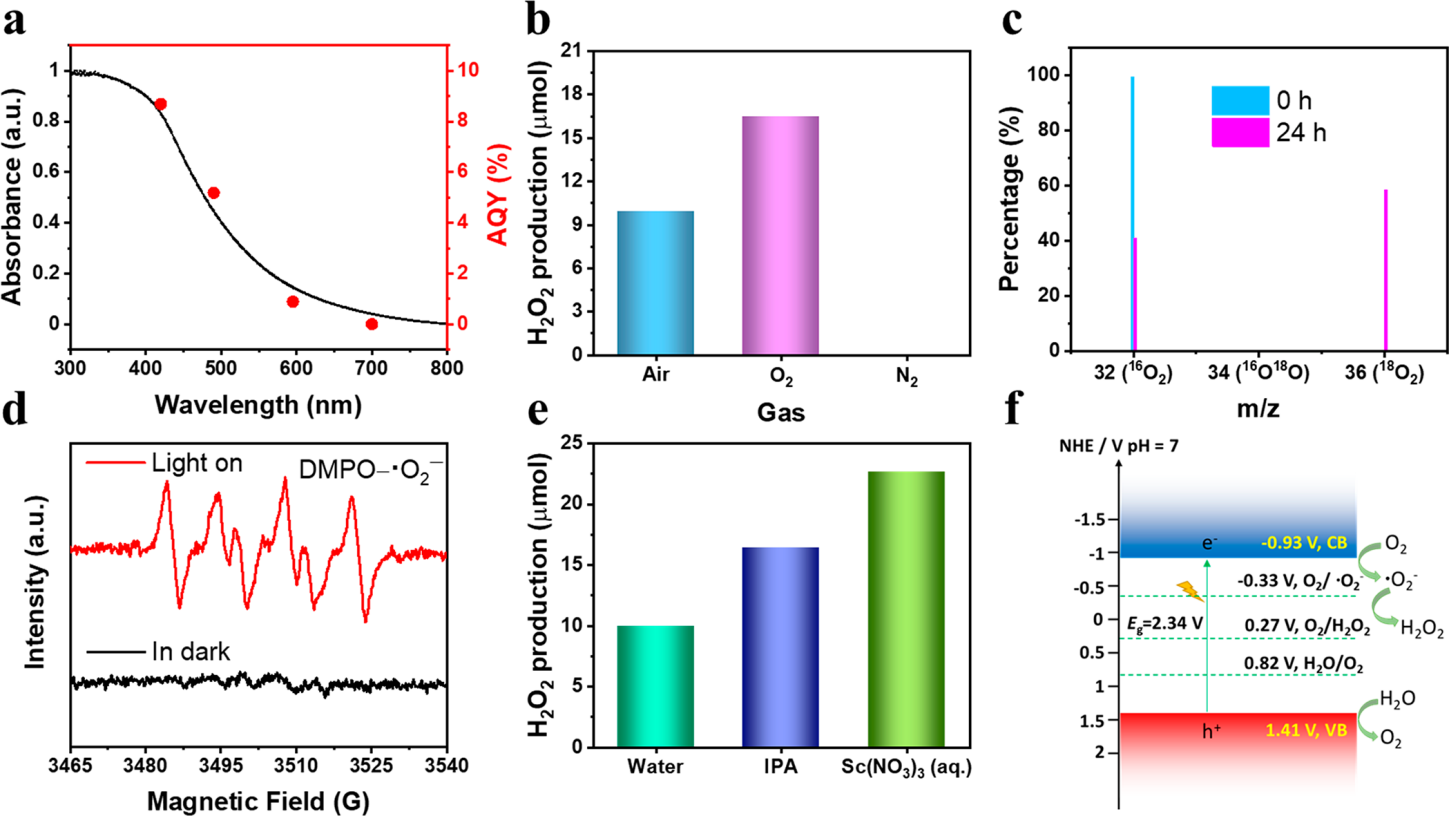
(a) Wavelength-dependent AQE values (measured in the first 1 h) and solid-state UV–visible spectrum of DE7-M. (b) Reactions under different gas atmospheres: 5 mg of DE7-M in 3 mL of H_2_O, 1.5 h (solar simulator). (c) Isotopic ^18^O_2_ labeling experiments with ^18^O_2_. The signal of ^16^O_2_ was from air during GC-MS injection. (d) EPR trapping experiments in the presence of DMPO as an electron-trapping agent. (e) Photocatalytic H_2_O_2_ production for DE7-M in neat water, with IPA (5 mg of DE7-M, 2.7 mL of H_2_O, 0.3 mL of IPA) and Sc(NO_3_)_3_ (150 mM aqueous solution, 5 mg of DE7-M, 3 mL), 1.5 h (solar simulator). (f) Measured energy band positions and proposed photocatalytic mechanism for DE7-M.

The H_2_O_2_ production was quenched when the **^•^**O_2_^–^ scavenger *p*-benzoquinone (*p*-BQ)^22^ was added. In the presence of Sc(NO_3_)_3_ (a strong Lewis acid),^23^ the H_2_O_2_ production performance of DE7-M was significantly enhanced (Figure 3e), which we attribute to the inhibition of back electron transfer from **^•^**O_2_^–^ after binding of Sc^3+^ to **^•^**O_2_^–^.^8^ On the basis of these results, we suggest that the photoinduced H_2_O_2_ production of DE7-M involves the stepwise reduction of O_2_.^3^

To gain deeper insight into the mechanism, we explored the half-reaction of DE7-M for H_2_O_2_ production by the addition of sacrificial electron-donor agents (Figure 3e and Figure S21). The increase in rate in the presence of isopropanol (IPA) suggests that H_2_O_2_ production might not be occurring via water oxidation by photoinduced holes,^10^ since these would be consumed by such electron donors. No H_2_O_2_ was detected when alkaline sacrificial reagents were used (Figure S22), probably because of decomposition of H_2_O_2_.^24^ AgNO_3_ also functioned as an electron-acceptor sacrificial agent in the half-reaction for O_2_ production (Figure S23), indicating that DE7-M can oxidize H_2_O to O_2_. The conduction band (CB) and valence band (VB) levels for DE7-M were estimated to be −0.93 V (vs NHE) and 1.41 V (vs NHE), which suggests that reduction and oxidation of oxygen and water are thermodynamically possible (Figure 3f and Figure S24). On the basis of these various results, we propose a mechanism for photocatalytic H_2_O_2_ production by DE7-M (Figure 3f).

The experiments discussed so far relate to short reaction times (<5 h). To be practically useful, long-term photostability of catalysts is essential. We therefore tested the photostability of DE7-M. First, the DE7-M material was used in five sequential 2 h photocatalytic H_2_O_2_ production runs (Figure 4a). The activity was found to decline over the last four runs, and there was dramatic catalyst mass loss, suggesting decomposition. In a continuous, 55.5 h experiment (Figure 4b), the H_2_O_2_ production rate leveled off after about 30 h. H_2_O_2_ consumption (decomposition) started to occur after 47.5 h, suggesting that the reaction had ceased (Figure S25). Similar photocatalytic H_2_O_2_ production profiles were observed for composites of procyanidins-methoxybenzaldehyde (PM) dipolymers with carbon dots.^25^ Bubbling fresh O_2_ into the reactor did not recover the production rate (Figure 4b). For comparison, we also tested the published resorcinol formaldehyde catalyst RF523, which we synthesized using the reported conditions (Figure S26).^11^ RF523 was found to be significantly less active than DE7-M at short reaction times under these conditions (Figure 4b), but it, too, became deactivated over time (Figure 4b) and underwent chemical changes (Figure S26c); again, the H_2_O_2_ production rate tended to zero after about 50 h of irradiation. Previously, carbon nitrides have shown good stability,^21,26^ but their catalytic activity is generally low for photocatalytic H_2_O_2_ production from pure water, which may in part account for this.

**Figure 4:**
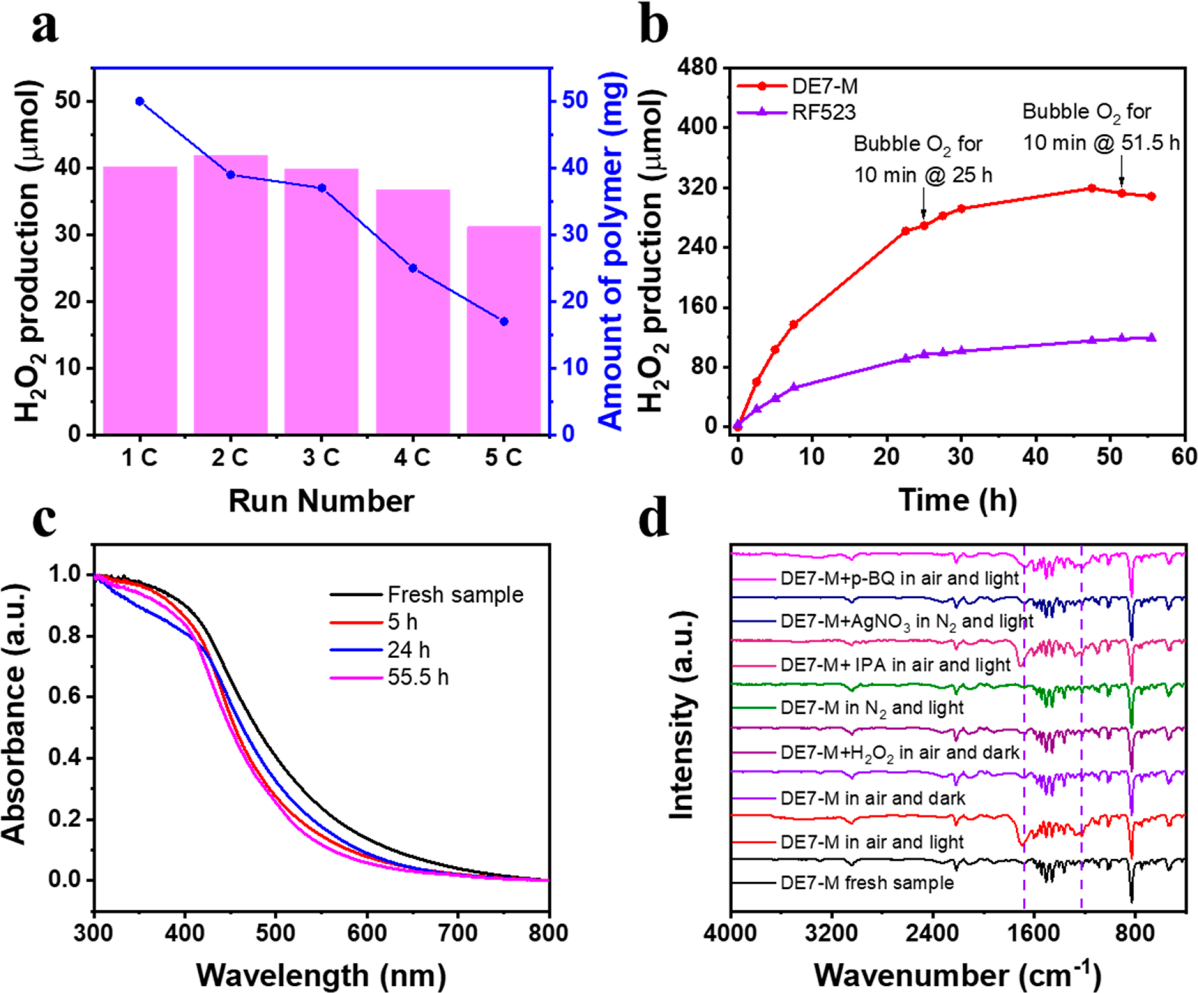
(a) Five sequential 2 h photocatalytic H_2_O_2_ production runs for DE7-M under >420 nm illumination. (b) Long-term photocatalytic H_2_O_2_ production of DE7-M and RF523 under >420 nm illumination. (c) Solid UV–vis spectra of fresh DE7-M and DE7-M after 5, 24, and 55.5 h of reaction. (d) FTIR spectra of DE7-M in different reaction conditions. Light source: solar simulator.

The morphology of DE7-M was maintained over the first 24 h, but after 55.5 h of irradiation, the particle size was notably reduced (Figure S27). Also, the absorption spectrum of DE7-M became blue-shifted after 55.5 h of irradiation (Figure 4c). PXRD patterns (Figure S28) and the elemental composition (Table S3) also changed. FTIR spectra showed a growing peak at 1672 cm^–1^, which was assigned to carbonyl functionalities, suggesting oxidation of DE7-M (Figure S29). Further analysis showed that the alkyne bonds in DE7-M are oxidized, ultimately to produce 1,4-terphthalic acid and 2,5-pyridinedicarboxylic acid (Figure S30a).^27^

There are at least two possible pathways for the degradation of DE7-M: the polymer could be attacked by **^•^**O_2_^–^ produced via photoinduced one-electron reduction of O_2_, or it could be self-oxidized by photoinduced holes.^11^ To study this, a series of control experiments was carried out (Figure 4d). There was no observable change in the FTIR for DE7-M under dark conditions in the presence of H_2_O/air or H_2_O_2_/air. DE7-M was not inherently unstable to H_2_O_2_ at these concentrations (∼3 mM). The polymer also remained intact after illumination under a nitrogen atmosphere. In the presence of IPA (a hole scavenger), both the rate of H_2_O_2_ production and the polymer decomposition rate were increased significantly (Figure S31), indicating that water oxidation is the slow step during photoinduced H_2_O_2_ production. This also suggested that photoinduced holes in the polymer are not the main cause of the decomposition since these holes might be expected to be scavenged by IPA. In support of this, DE7-M neither decomposed nor produced H_2_O_2_ in the presence of AgNO_3_, an electron scavenger (Figure 4d). In the presence of *p-*BQ, an **^•^**O_2_^–^ scavenger, the decomposition of DE7-M was significantly mitigated (Figure 4d). Taken together, these data suggest that the main pathway for DE7-M decomposition involves **^•^**O_2_^–^ (Figure S30b).^27^

We next measured the AQY in the presence of IPA, which reflects the intrinsic efficiency of the photoinduced exciton generation, separation, and migration; this was found to be 20.4% at 420 nm, highlighting the potential for DE7-M or similar materials as photocatalysts, providing that decomposition pathways can be suppressed. One strategy might be to load cocatalysts onto the surface of DE7-M, as exemplified in photocatalytic overall water splitting,^28,29^ to promote O_2_ activation to H_2_O_2_^30,31^ and to accelerate the H_2_O oxidation reaction,^32,33^ respectively. Cocatalysts might also suppress reactions between the polymer and photoinduced electrons and holes,^34,35^ providing that they do not also catalyze H_2_O_2_ decomposition.^36,37^ Another strategy would be to develop materials that are intrinsically less susceptible to **^•^**O_2_^–^ attack or to adjust the band energy positions to produce H_2_O_2_ via a one-step, two-electron reduction pathway, avoiding the production of ^•^O^2–^ intermediates.^10,13^

In summary, a conjugated polymer, poly(3–4-ethynylphenyl)ethynyl)pyridine, DE7, is an efficient organic photocatalyst for H_2_O_2_ production from H_2_O and O_2_ under visible light illumination without added sacrificial agents. DE7 outperforms many other organic catalysts over short reaction times. However, DE7 also decomposes over quite short irradiation time scales, rendering practical applications impossible at this stage. Similar deactivation was observed for a resorcinol formaldehyde resin, RF523.^11^ This suggests that more attention must be paid to catalyst stability, as well as increasing photocatalytic rates. Given that longer-term measurements are relatively straightforward, we would suggest that kinetic experiments of at least 24 h duration, and preferably longer (Figure 4b), should be mandatory for new catalysts.

## Supporting Information Available

Materials and methods, experimental details, Supporting Figures S1–S31, and Supporting Tables S1–S3 (PDF)
