# Supplementary material for: Linear Conjugated Polymers for Solar-Driven Hydrogen Peroxide Production: The Importance of Catalyst Stability
Source: J Am Chem Soc. 2021 Nov 10;143(46):19287–93. doi: 10.1021/jacs.1c09979 (PMC8630703; doi:10.1021/jacs.1c09979)
Supplement: Supplementary file 1 — ja1c09979_si_001.pdf [file ja1c09979_si_001.pdf]

Electronic Supporting Information

Linear Conjugated Polymers for Solar-Driven Hydrogen Peroxide Production: The Importance of Catalyst Stability

Lunjie Liu,[†] Mei-Yan Gao,[‡] Haofan Yang,[†] Xiaoyan Wang,[†] Xiaobo Li[†*] and Andrew I. Coope[†*]

[†] Department of Chemistry and Materials Innovation Factory, University of Liverpool, 51 Oxford Street, Liverpool, L7 3NY, United Kingdom.

[‡] Department of Chemical Sciences, Bernal Institute, University of Limerick, Limerick, V94 T9PX, Republic of Ireland.

Correspondence emails: aicooper@liverpool.ac.uk； Xiaobo.Li@liverpool.ac.uk.

Contents

1. Materials .... - 1 -
2. Characterization Methods .... - 1 -
3. Synthesis procedures .... - 2 -
3.1 General synthesis procedure for TA-n COFs[1] .... - 2 -
3.2 General synthesis procedure for PY/TE-OF-n and DE7 derivatives .... - 3 -
3.3 Synthesis procedure for DE7-M .... - 7 -
3.4 Synthesis procedure for RF523 .... - 7 -
4. Photocatalytic experiments .... - 8 -
4.1 High-throughput photocatalytic H_2O_2 production experiment .... - 8 -
4.2 Kinetic H_2O_2 production experiment .... - 8 -
5. Photocatalytic conversion efficiency .... - 9 -
5.1 AQY measurement .... - 9 -
5.2 SCC Efficiency measurement .... - 9 -
6. Electrochemical analysis .... - 10 -
6.1 Photocurrent response and impedance .... - 10 -
6.2 Mott-Schottky measurement .... - 10 -
7. Electron paramagnetic resonance measurements .... - 10 -
8. Isotopic exchange experiments .... - 11 -
9. Supporting figures .... - 12 -
Figure S1: UV-vis spectra and IR spectra of TA-n COFs .... - 12 -
Figure S2: UV-vis spectra and IR spectra of DD-n SMs .... - 12 -
Figure S3: UV-vis spectra and IR spectra of PY/TE-OF-n CMPs .... - 13 -
Figure S4: UV-vis spectra and IR spectra of DE7 and DE7 derivatives ...... - 13 -
Figure S5: PXRD patterns and TGA data of DE7 and DE7 derivatives .... - 14 -
Figure S6: SEM images of DE7 and DE7 derivatives .... - 14 -
Figure S7: Images of contact angle against water for DE7 and DE7 derivatives .... - 15 -

Figure S8: Property-performance relationships for DE7 and DE7 derivatives .... - 15 -
Figure S9: Photocatalytic H_2 and H_2O_2 production of DE-n and TE-n polymers .... - 16 -
Figure S10: Synthesis procedures for DE7 and DE7-M .... - 16 -
Figure S11: Solid-state NMR spectra .... - 17 -
Figure S12: Raman spectra .... - 17 -
Figure S13: XPS spectra .... - 18 -
Figure S14: Thermogravimetric data .... - 19 -
Figure S15: Brunauer-Emmett-Teller surface area .... - 19 -
Figure S16: Effect of residual Pd and Cu .... - 20 -
Figure S17: Photocatalytic H_2O_2 production of DE7 and DE7-M .... - 21 -
Figure S18: Optoelectronic properties of DE7 and DE7-M .... - 21 -
Figure S19: Contact angle measurements .... - 22 -
Figure S20: Solar-to-chemical conversion (SCC) efficiency .... - 22 -
Figure S21: Half photoreaction of DE7-M for H_2O_2 production................................... - 23 -
Figure S22: Photocatalytic H_2O_2 production of DE7-M at different pH .... - 23 -
Figure S23: Photocatlaytic water oxidation half reaction for DE7-M............................ - 24 -
Figure S24: Electrochemical Mott-Schottky measurements... - 24 -
Figure S25: Photocatalytic decomposition of H_2O_2.. - 25 -
Figure S26: SEM images, UV-vis spectrum and FT-IR spectra for RF523 .... - 26 -
Figure S27: SEM images of DE7-M after long-term photoreaction.............................. - 27 -
Figure S28: PXRD patterns of DE7-M after long-term photoreaction........................... - 27 -
Figure S29: FT-IR spectra of DE7-M after long-term photoreaction............................. - 28 -
Figure S30: Proposed decomposition route for DE7-M .... - 29 -
Figure S31: Long-term photoreaction of DE7-M with the addition of IPA .... - 30 -
10. Supporting tables .... - 31 -
Table S1: Cu contents of polymers synthesized via Sonogashira coupling reactions .... - 31 -

Table S2: Comparation of the catalytic H_2O_2 production activity of DE7-M with other reported organic materials[a] ... - 32 -

Table S3: Elemental analysis results of DE7-M after long-term photoreaction ... - 33 -

11. References ... - 34 -

1. Materials

All reagents were obtained from Sigma-Aldrich, Alfa Aesar and Fluorochem, and used as received.

2. Characterization Methods

The absorption spectra for the polymers were recorded on a Cary 5000 UV-visible-NIR spectrophotometer as powders. Transmission FT-IR spectra were obtained with an attenuated total reflectance (ATR) method on a Bruker Tensor-27 spectrometer at room temperature. Photoluminescence spectra were performed on a Shimadzu RF-5301PC fluorescence spectrometer. Time-correlated single photon counting (TCSPC) experiments were measured on an Edinburgh Instruments LS980-D2S2-STM spectrometer (EPL-375 diode, $\lambda = 371$ nm). The instrument response of the TCSPC spectrometer was measured with colloidal silica (LUDOX HS-40, Sigma-Aldrich) at the excitation wavelength. Decay times were fitted in the FAST software using three decay exponents. Thermogravimetric analysis was performed by heating samples under air in open platinum pans from 25 to 1000 °C at 10 °C min^{-1} on an EXSTAR6000 instrument. PXRD measurements were performed on a Panalytical Empyrean diffractometer with a Cu X-ray source. Static light scattering measurements were performed on a Malvern Mastersizer 3000 Particle Sizer at a laser obscuration of 5-10%. Particle sizes were fitted according to the Mie theory with the Malvern 'General Purpose' analysis model. Surface areas were tested on Micromeritics 2420 instrument at a temperature of 77 K. Before analysis, polymer samples were degassed offline at 110 °C for 15 hours under dynamic vacuum (10^{-5} bar). CHNS-O Analyzer was measured using standard microanalytical procedures. Palladium, platinum and copper contents were determined using ICP-OES Agilent 5110 equipped with a collision/reaction cell after a microwave digestion of the materials in nitric acid (67-69%, trace metal analysis grade) in a microwave. Water contact angles were measured using pressed pellets and a drop-shape analysis apparatus (Krüss DSA100) with the Young-Laplace fitting method. The morphology of the polymers was studied using a Hitachi S4800 SEM and Tecnai F20 FE-TEM. X-Ray photoelectron spectroscopy (XPS) measurements were performed on a Thermo Fisher ESCALAB 250Xi instrument. The Raman measurements were performed on an inVia Reflex Qontor Confocal Raman microscope upon excitation of 785 nm laser. ^{13}C Magic-angle spinning measurements were carried out at 100.63 MHz using a Bruker Avance III HD spectrometer and 4 mm (rotor o.d.) probe. Spectra were acquired at a spin rate

of 10 kHz. Cross-polarisation (CP) spectra were recorded with TOSS spinning sideband suppression, 4 ms contact time and with a recycle delay of 4 s. Carbon spectral referencing is relative to neat tetramethylsilane, carried out by setting the high frequency signal from an external sample of adamantane to 38.5 ppm.

3. Synthesis procedures

3.1 General synthesis procedure for TA-n COFs[1]

A Pyrex tube was charged with monomers, mesitylene (0.5 mL), 1,4-dioxane (0.5 mL) and 6 M acetic acid (0.1 mL); the mixture was then degassed by three freeze-pump-thaw cycles. After sealing and heating at 120 °C for 3 days in an oven, the precipitate was collected by filtration and washed with anhydrous tetrahydrofuran and acetone. The final product was dried at 100 °C under vacuum overnight.

TA1: Terephthalaldehyde (20 mg, 0.15 mmol) and 4,4',4"-(1,3,5-triazine-2,4,6-triyl)trianiline (35 mg, 0.1 mmol) and were used in this reaction described in the general procedure. After work-up and washing, the product was obtained as an orange powder (21 mg, 43%). Anal. Calcd for $(C_{32}H_{20}N_5)n$: C, 80.99; H, 4.25; N, 14.76%. Found: C, 74.35; H, 4.36; N, 16.50%.

TA2: 2,5-Dihydroxyterephthalaldehyde (25 mg, 0.15 mmol) and 4,4',4"-(1,3,5-triazine-2,4,6-triyl)trianiline (35 mg, 0.1 mmol) and were used in this reaction described in the general procedure. After work-up and washing, the product was obtained as a red solid (44 mg, 84%). Anal. Calcd for $(C_{32}H_{20}N_5O_3)n$: C, 73.55; H, 3.86; N, 13.40%. Found: C, 69.58; H, 4.04, N, 13.74%.

TA3: 1,5-Naphthalenedicarboxaldehyde (28 mg, 0.15 mmol) and 4,4',4"-(1,3,5-triazine-2,4,6-triyl)trianiline (35 mg, 0.1 mmol) and were used in this reaction described in the general procedure. After work-up and washing, the product was obtained as a crimson solid (52 mg, 95%). Anal. Calcd for $(C_{38}H_{23}N_5)n$: C, 83.04; H, 4.22; N, 12.74%. Found: C, 71.14; H, 4.03, N, 12.29%.

TA4: 4,4'-Biphenyldicarboxaldehyde (32 mg, 0.15 mmol) and 4,4',4''-(1,3,5-triazine-2,4,6-triyl)trianiline (35 mg, 0.1 mmol) and were used in this reaction described in the general procedure. After work-up and washing, the product was obtained as an orange solid (27 mg, 46%). Anal. Calcd for $(C_{41}H_{26}N_5)n$: C, 83.65; H, 4.45, N, 11.90%. Found: C, 72.73; H, 4.75; N, 18.33%.

TA5: 4,4',4''-(1,3,5-Triazine-2,4,6-triyl)tribenzaldehyde (17 mg, 0.04 mmol) and 4,4',4''-(1,3,5-triazine-2,4,6-triyl)trianiline (15 mg, 0.04 mmol) and were used in this reaction described in the general procedure. After work-up and washing, the product was obtained as a yellow solid (24 mg, 81%). Anal. Calcd for $(C_{24}H_{15}N_6)_n$: C, 77.91; H, 3.92; N, 18.17%. Found: C, 76.73; H, 4.05; N, 17.53%.

3.2 General synthesis procedure for PY/TE-OF-n and DE7 derivatives

A 40 mL glass vial was charged with the monomers, $Pd(PPh_3)_4$, CuI, anhydrous *N*,*N*-dimethylformamide, and triethylamine, and then sealed with a silicone septum in a glovebox. The sealed vessels were taken out of the glovebox, inserted in a sand bath preheated to 100 °C and kept at this temperature for 2 days. After cooling to room temperature, the mixture was quenched by addition of methanol, and the solids were filtered off and washed with methanol and acetone. Further purification was carried out by Soxhlet extraction with chloroform for 2 days. The final product was dried in the vacuum oven at 80 °C overnight. For TE-n and DE-n polymers, the detail process can be seen in previous work.[2]

PY-OF1: 1,4-Dibromo-2,5-dimethoxybenzene (118 mg, 0.4 mmol), 1,3,6,8-tetraethynylpyrene (60 mg, 0.2 mmol), $Pd(PPh_3)_4$ (15 mg, 0.01 mmol), CuI (10 mg, 0.05 mmol), *N*,*N*-dimethylformamide (9 mL) and triethylamine (9 mL) were used in this Sonogashira polycondensation reaction described in the general procedure. After work-up and Soxhlet, the product was obtained as a red solid (113 mg, 82%). Anal. Calcd for $(C_{40}H_{22}O_4)_n$: C, 84.79; H, 3.91; Found C, 75.78; H, 3.58. Pd content: 1.41%, Cu content: 0.47%.

PY-OF2: 2,5-Dibromo-1,4-benzoquinone (106 mg, 0.4 mmol), 1,3,6,8-tetraethynylpyrene (60 mg, 0.2 mmol), $Pd(PPh_3)_4$ (15 mg, 0.01 mmol), CuI (10 mg, 0.05 mmol), *N*,*N*-dimethylformamide (9 mL) and triethylamine (9 mL) were used in this Sonogashira polycondensation reaction described in the general procedure. After work-up and Soxhlet, the product was obtained as a red solid (97 mg, 95%). Anal. Calcd for $(C_{36}H_{10}O_4)_n$: C, 85.37; H, 1.99; Found C, 72.03; H, 3.49. Pd content: 0.69%, Cu content: 0.28%.

PY-OF3: 2,5-Dibromoterephthalic acid (130 mg, 0.4 mmol), 1,3,6,8-tetraethynylpyrene (60 mg, 0.2 mmol), $Pd(PPh_3)_4$ (15 mg, 0.01 mmol), CuI (10 mg, 0.05 mmol), *N*,*N*-dimethylformamide (9 mL) and triethylamine (9 mL) were used in this Sonogashira polycondensation reaction described in the general procedure. After work-up and Soxhlet, the product was obtained as a red solid (125 mg, 86%). Anal. Calcd for $(C_{40}H_{14}O_8)_n$: C, 77.17; H, 2.27; Found C, 69.30; H, 3.79. Pd content: 0.96%, Cu content: 1.24%.

PY-OF4: 2,5-Dibromohydroquinone (107 mg, 0.4 mmol), 1,3,6,8-tetraethynylpyrene (60 mg, 0.2 mmol), $Pd(PPh_3)_4$ (15 mg, 0.01 mmol), CuI (10 mg, 0.05 mmol), *N*,*N*-dimethylformamide (9 mL) and triethylamine (9 mL) were used in this Sonogashira polycondensation reaction described in the general procedure. After work-up and Soxhlet, the product was obtained as a red solid (102 mg, 88%). Anal. Calcd for $(C_{36}H_{14}O_4)_n$: C, 84.70; H, 2.76; Found C, 75.86; H, 3.51. Pd content: 1.07%, Cu content: 1.03%.

TE-OF5: 2,4,6-Tribromophenol (331 mg, 1 mmol), 1,3,5-Triethynylbenzene (150 mg, 1 mmol), $Pd(PPh_3)_4$ (18 mg, 0.01 mmol), CuI (10 mg, 0.05 mmol), *N*,*N*-dimethylformamide (9 mL) and triethylamine (9 mL) were used in this Sonogashira polycondensation reaction described in the general procedure. After work-up and Soxhlet, the product was obtained as a yellow solid (215 mg, 90%). Anal. Calcd for $(C_{14}H_6O)_n$: C, 88.88; H, 2.66; O, 8.46. Found C, 69.74; H, 3.45. Pd content: 0.37%, Cu content: 0.23%.

TE-OF6: 2,4,6-Tribromophenol (347 mg, 1 mmol), 1,3,5-Triethynylbenzene (150 mg, 1 mmol), $Pd(PPh_3)_4$ (18 mg, 0.01 mmol), CuI (10 mg, 0.05 mmol), *N*,*N*-dimethylformamide (9

mL) and triethylamine (9 mL) were used in this Sonogashira polycondensation reaction described in the general procedure. After work-up and Soxhlet, the product was obtained as a yellow solid (221 mg, 87%). Anal. Calcd for $(C_{14}H_6O_2)_n$: C, 81.95; H, 2.46; O, 15.59. Found C, 69.62; H, 3.74. Pd content: 0.62%, Cu content: 0.25%.

TE-OF7: 2,4,6-Tribromophenol (363 mg, 1 mmol), 1,3,5-Triethynylbenzene (150 mg, 1 mmol), $Pd(PPh_3)_4$ (18 mg, 0.01 mmol), CuI (10 mg, 0.05 mmol), *N*,*N*-dimethylformamide (9 mL) and triethylamine (9 mL) were used in this Sonogashira polycondensation reaction described in the general procedure. After work-up and Soxhlet, the product was obtained as a yellow solid (219 mg, 81%). Anal. Calcd for $(C_{14}H_6O_3)_n$: C, 76.02; H, 2.28; O, 21.70. Found C, 71.69; H, 2.61. Pd content: 0.48%, Cu content: 0.14%.

DE7: 2,5-Dibromopyridine (237 mg, 1 mmol), 1,4-diethynylbenzene (126 mg, 1 mmol), $Pd(PPh_3)_2Cl_2$ (18 mg, 0.025 mmol), CuI (2 mg, 0.010 mmol), triphenylphosphine (13 mg, 0.050 mmol), *N*,*N*-dimethylformamide (9 mL) and triethylamine (9 mL) were used in this Sonogashira polycondensation reaction described in the general procedure. After work-up and Soxhlet the product was obtained as a yellow solid (193 mg, 96%). Anal. Calcd for $(C_{15}H_7N)_n$: C, 89.53; H, 3.51; N, 6.96%. Found: C, 76.33; H, 3.28; N, 5.42%. Pd content: 0.27%, Cu content: 0.02%.

DE7-D1: 1,4-Dibromobenzene (236 mg, 1 mmol), 1,4-diethynylbenzene (126 mg, 1 mmol), $Pd(PPh_3)_2Cl_2$ (18 mg, 0.025 mmol), CuI (2 mg, 0.010 mmol), triphenylphosphine (13 mg, 0.050 mmol), *N*,*N*-dimethylformamide (9 mL) and triethylamine (9 mL) were used in this Sonogashira polycondensation reaction described in the general procedure. After work-up and Soxhlet the product was obtained as a yellow solid (191 mg, 95%). Anal. Calcd for $(C_{16}H_8)_n$: C, 95.97; H, 4.03%. Found: C, 82.44; H, 3.67%. Pd content: 0.58%, Cu content: 0.01%.

DE7-D2: 2,4-Dibromopyridine (237 mg, 1 mmol), 1,4-diethynylbenzene(126 mg, 1 mmol), $Pd(PPh_3)_2Cl_2$ (18 mg, 0.025 mmol), CuI (2 mg, 0.010 mmol), triphenylphosphine (13 mg, 0.050 mmol), *N*,*N*-dimethylformamide (9 mL) and triethylamine (9 mL) were used in this

Sonogashira polycondensation reaction described in the general procedure. After work-up and Soxhlet the product was obtained as a yellow solid (186 mg, 92%). Anal. Calcd for $(C_{15}H_7N)_n$: C, 89.53; H, 3.51; N, 6.96%. Found: C, 79.95; H, 3.28; N, 5.87%. Pd content: 0.81%, Cu content: 0.01%.

DE7-D3: 2,6-Dibromopyridine (237 mg, 1 mmol), 1,4-diethynylbenzene(126 mg, 1 mmol), $Pd(PPh_3)_2Cl_2$ (18 mg, 0.025 mmol), CuI (2 mg, 0.010 mmol), triphenylphosphine (13 mg, 0.050 mmol), *N*,*N*-dimethylformamide (9 mL) and triethylamine (9 mL) were used in this Sonogashira polycondensation reaction described in the general procedure. After work-up and Soxhlet the product was obtained as a yellow solid (191 mg, 95%). Anal. Calcd for $(C_{15}H_7N)_n$: C, 89.53; H, 3.51; N, 6.96%. Found: C, 78.78; H, 3.25; N, 5.77%. Pd content: 1.02%, Cu content: 0.02%.

DE7-D4: 3,5-Dibromopyridine (237 mg, 1 mmol), 1,4-diethynylbenzene(126 mg, 1 mmol), $Pd(PPh_3)_2Cl_2$ (18 mg, 0.025 mmol), CuI (2 mg, 0.010 mmol), triphenylphosphine (13 mg, 0.050 mmol), *N*,*N*-dimethylformamide (9 mL) and triethylamine (9 mL) were used in this Sonogashira polycondensation reaction described in the general procedure. After work-up and Soxhlet the product was obtained as a yellow solid (194 mg, 97%). Anal. Calcd for $(C_{15}H_7N)_n$: C, 89.53; H, 3.51; N, 6.96%. Found: C, 76.98; H, 3.21; N, 5.63%. Pd content: 0.81%, Cu content: 0.03%.

DE7-D5: 4,7-Dibromobenzo[c]-1,2,5-thiadiazole (294 mg, 1 mmol), 1,4-diethynylbenzene(126 mg, 1 mmol), $Pd(PPh_3)_2Cl_2$ (18 mg, 0.025 mmol), CuI (2 mg, 0.010 mmol), triphenylphosphine (13 mg, 0.050 mmol), *N*,*N*-dimethylformamide (9 mL) and triethylamine (9 mL) were used in this Sonogashira polycondensation reaction described in the general procedure. After work-up and Soxhlet the product was obtained as a orange solid (249 mg, 96%). Anal. Calcd for $(C_{16}H_6N_2S)_n$: C, 74.40; H, 2.34; N, 10.85; S, 12.41%. Found: C, 68.38; H, 2.49; N, 9.61; S, 11.18%. Pd content: 0.73%, Cu content: 0.01%.

DE7-D6: 5,8-Dibromoquinoxaline (288 mg, 1 mmol), 1,4-diethynylbenzene(126 mg, 1 mmol), $Pd(PPh_3)_2Cl_2$ (18 mg, 0.025 mmol), CuI (2 mg, 0.010 mmol), triphenylphosphine (13 mg, 0.050 mmol), *N*,*N*-dimethylformamide (9 mL) and triethylamine (9 mL) were used in this Sonogashira polycondensation reaction described in the general procedure. After work-up and Soxhlet the product was obtained as a brown solid (244 mg, 97%). Anal. Calcd for $(C_{18}H_8N_2)_n$: C, 85.70; H, 3.20; N, 11.10%. Found: C, 75.44; H, 3.26; N, 9.23%. Pd content: 0.63%, Cu content: 0.03%.

DE7-D7: 4,7-Dibromobenzo[c][1,2,5]oxadiazole (278 mg, 1 mmol), 1,4-diethynylbenzene(126 mg, 1 mmol), $Pd(PPh_3)_2Cl_2$ (18 mg, 0.025 mmol), CuI (2 mg, 0.010 mmol), triphenylphosphine (13 mg, 0.050 mmol), *N*,*N*-dimethylformamide (9 mL) and triethylamine (9 mL) were used in this Sonogashira polycondensation reaction described in the general procedure. After work-up and Soxhlet the product was obtained as a red solid (238 mg, 98%). Anal. Calcd for $(C_{16}H_6N_2O)_n$: C, 79.33; H, 2.50; N, 11.56%. Found: C, 73.61; H, 2.68; N, 9.72%. Pd content: 0.65%, Cu content: 0.01%.

3.3 Synthesis procedure for DE7-M

A glass vial was charged with 2,5-dibromopyridine (1 mmol), 1,4-diethynylbenzene (1 mmol), $Pd(PPh_3)_2Cl_2$ (0.025 mmol), CuI (0.010 mmol), triphenylphosphine (0.050 mmol), *N*,*N*-dimethylformamide (9 mL) and triethylamine (9 mL), and then sealed with a silicone septum in a glovebox. The sealed vessels were taken out of the glovebox and placed in a microwave chamber heated to 80 °C for 2 h. After reaction, the mixture was quenched by addition of methanol. The solids were filtered off and washed with methanol and acetone. Further purification was carried out by Soxhlet extraction with chloroform for 2 days. The final product was dried in the vacuum oven at 80 °C overnight. The final product was obtained as a yellow solid(196 mg, 97%). Anal. Calcd for $(C_{15}H_7N)_n$: C, 89.53; H, 3.51; N, 6.96%. Found: C, 74.98; H, 3.50; N, 6.85%. Pd content: 0.24%, Cu content: 0.02%.

3.4 Synthesis procedure for RF523

Here, we followed a published route.[3] Resorcinol (3.6 mmol), formaldehyde (37 wt.% solution, 7.2 mmol) and NH_3 (28 wt.% solution, 3.0 mmol) were added to deionized water (40 ml) and

stirred for 5 min at room temperature. The white colloidal suspension was transferred to a Teflon-lined stainless-steel autoclave and left in an oven, where the heating rate was 7 K min^{-1} and the holding time at 523 K was 24 h. The solids formed were washed thoroughly by Soxhlet extraction with acetone for 12 h and dried in vacuo at room temperature for 12 h.

4. Photocatalytic experiments

4.1 High-throughput photocatalytic H_2O_2 production experiment

A sample vial was charged with polymer powder (5 mg) and either water (3 mL) or water (2.7 mL) plus a sacrificial reagent (0.3 mL), and then ultrasonicated for 10 min (to disperse the polymes) after being capped under air. The photocatalytic H_2O_2 evolution experiments were performed on an Oriel Solar Simulator 94123A with an output of 1.0 sun (Class AAA, 1440 W xenon, 12 × 12 in.). After 1.5 h, 1 mL solution was sampled with an injection syringe after shook evenly and then filtered with a 0.2 μm Millipore filter to remove the photocatalyst. The amount of H_2O_2 produced was analysed with Peroxide test sticks (for semi-quantitative initial screening) or a KI titrimetric method[4] (for quantification).

4.2 Kinetic H_2O_2 production experiment

A flask was charged with 50 mg of polymer (DE7-M or RF523) powders and 30 mL water and sealed with a rubber septum. The suspension was ultrasonicated for 10 min to disperse well before degassing by O_2 bubbling for 10 min. The reaction solution was illuminated by a 300 W Xe lamp with a filter (λ > 420 nm) and was kept at room temperature by air cooling. The concentration of H_2O_2 was determined using a KI titrimetric method[4].

For recycle experiments, DE7-M was recovered every 2 hours by filtration and weighed between runs, and fresh water replaced.

5. Photocatalytic conversion efficiency

5.1 AQY measurement

The apparent quantum yield (AQY) was determined under monochromatic LED light irradiation at a certain wavelength (λ = 420 nm, 490 nm, 595 nm or 700 nm), and the light intensity was measured by a ThorLabs PM100D Power with a photodiode sensor.

The AQY was calculated using the following equation:

$$AQY\,\% = \frac{[\mathrm{H_2O_2\ produced\ (mol)}] \times 2}{\mathrm{photon\ number\ entered\ into\ the\ reactor\ (mol)}} \times 100$$

$$= \frac{[N_\mathrm{a} \times h \times c)][\mathrm{H_2O_2\ produced\ (mol)}] \times 2}{\mathrm{I \times S \times t \times \lambda}} \times 100$$

Where, N_a is Avogadro's constant (6.022×10^{23} mol^{-1}), h is the Planck constant (6.626×10^{-34} J s), c is the speed of light (3×10^{8} m s^{-1}), S is the irradiation area (cm^{2}), I is the intensity of irradiation light (W cm^{-2}), t is the photoreaction time (s), λ is the wavelength of the monochromatic light (m).

5.2 SCC Efficiency measurement

To determine the solar-to-chemical energy conversion (SCC) efficiency, a LED solar simulator was used as the light source (100 mW cm^{-2}). 200 mg catalysts and 50 mL water were added into a flask and bubbled with O_2 for 10 minutes, the reaction was carried out at 40 °C in a water bath. The SCC efficiency was calculated via following equation:

$$SCC\ efficiency\ (\%) =$$

$$\frac{[\ \Delta\mathrm{G\ for\ H_2O_2\ generation\ (J\ mol^{-1})}]\ [\mathrm{H_2O_2\ produced\ (mol)}]}{[\mathrm{total\ input\ energy\ (W)}]\ [\mathrm{reaction\ time\ (s)}]} \times 100$$

where the free energy (ΔG) for H_2O_2 formation is 117 kJ mol^{-1}, the irradiance of the spectrum is 1,000 W m^{-2} and the irradiated area is 3.14×10^{-4} m^{2}. The total input energy was therefore 0.314 W.

6. Electrochemical analysis

6.1 Photocurrent response and impedance

Electrochemical experiments were performed on a Bio-logic SP200 workstation. The transient photocurrent responses (*I*–t) and electrochemical impedance spectra (EIS) of samples were measured in a three-electrode system (sample on FTO as the working electrode, Pt plate as the counter electrode and Ag/AgCl as the reference electrode) under a 300 W Xe light source (Newport). The electrolyte was 0.5 M Na_2SO_4 aqueous solution. The working electrode was prepared as follows: 2 mg of the photocatalyst was ultrasonicated with 10 μL Nafion (5 wt.%) aqueous solution and 100 μL ethanol giving a slurry. The slurry was then coated onto FTO glass electrodes with an active area of 0.28 cm^2. The applied bias for intermittent photocurrent intensity measurement was 0.6 V *vs.* Ag/AgCl. The EIS spectra were determined by applying a 10 mV AC signal over the frequency range of 100 kHz to 10 mHz at a DC bias of 0.6 V *vs.* Ag/AgCl.

6.2 Mott-Schottky measurement

The Mott-Schottky plots were measured performed on a Bio-logic SP200 workstation in a standard three electrode system (working electrode : sample on FTO, counter electrode : Pt mesh, and reference electrode : Ag/AgCl) with 0.5 M Na_2SO_4 (pH = 7) as the electrolyte. Preparation of the working electrode: 2 mg polymer was added in 0.1 mL ethanol and 10 μL Nafion. After sonicated for 30 minutes, the slurry was deposited on the FTO substrate and dried in air.

7. Electron paramagnetic resonance measurements

Spin trapping electron paramagnetic resonance (EPR) measurements were performed using a an ESR spectrometer (Bruker-BioSpin, E500). 5,5-dimethyl-1-pyrroline N-oxide (DMPO) were used as a spin-trapping reagent to detect radicals. The measurements were carried out in a H_2O/methanol (1:9, 500 μL): mixture with 2 mg catalyst and 0.1 mmol DMPO, a Xe lamp with a filter ($\lambda > 420$ nm) was applied as the light source.

8. Isotopic exchange experiments

A vial was charged with 50 mg DE7-M and 35 mL $H_2{}^{16}O$ and sealed with a rubber septum, then bubbled with He for 10 min, $^{18}O_2$ gas (purity : 99%, ca. 10 mL) was subsequently injected by a syringe. The vial was illuminated by a Xe lamp with a filter (λ >420 nm) for 24 h, then He gas was bubbled again to remove the $^{18}O_2$ gas. After that, the reaction solution was injected into a vial containing MnO_2 and He gas, and the gas produced from the decomposition phase was detected by an Agilent 7890B GC-MS system. For the 0 h experiment (Figure 3c), exactly the same procedures were applied, except for the lack of irradiation. This shows that ^{18}O isotopically labelled H_2O_2 is produced in the irradiated case. The $^{16}O_2$ was from the air as the gas samples were injected into the GC with a syringe.

9. Supporting figures

Figure S1: UV-vis spectra and IR spectra of TA-n COFs

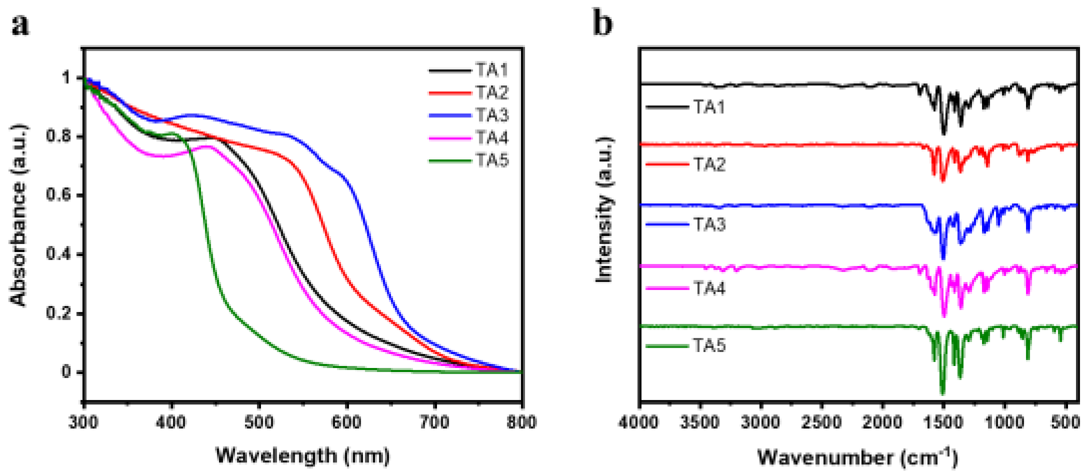

Figure S1: (a) UV-vis spectra and (b) IR spectra of TA1-5 COFs.

Figure S2: UV-vis spectra and IR spectra of DD-n SMs

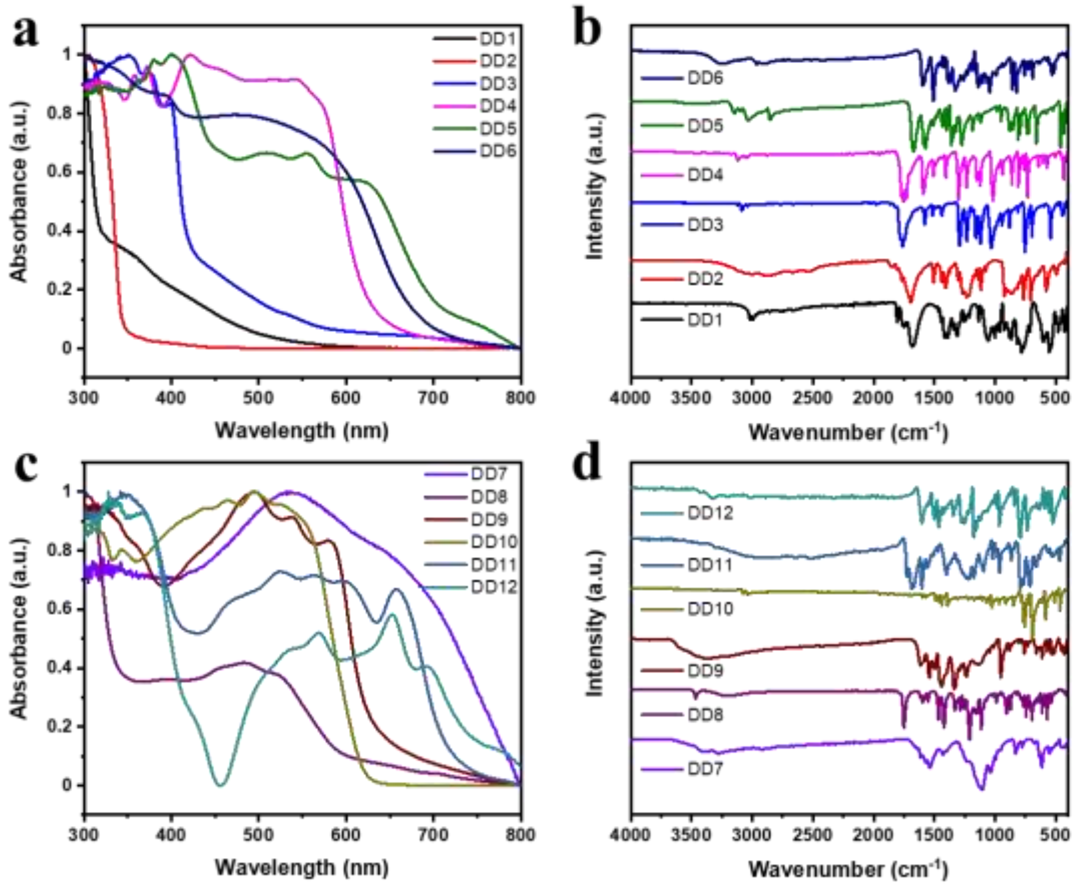

Figure S2: (a and c) UV-vis spectra and (b and d) IR spectra of DD1-12 SMs.

Figure S3: UV-vis spectra and IR spectra of PY/TE-OF-n CMPs

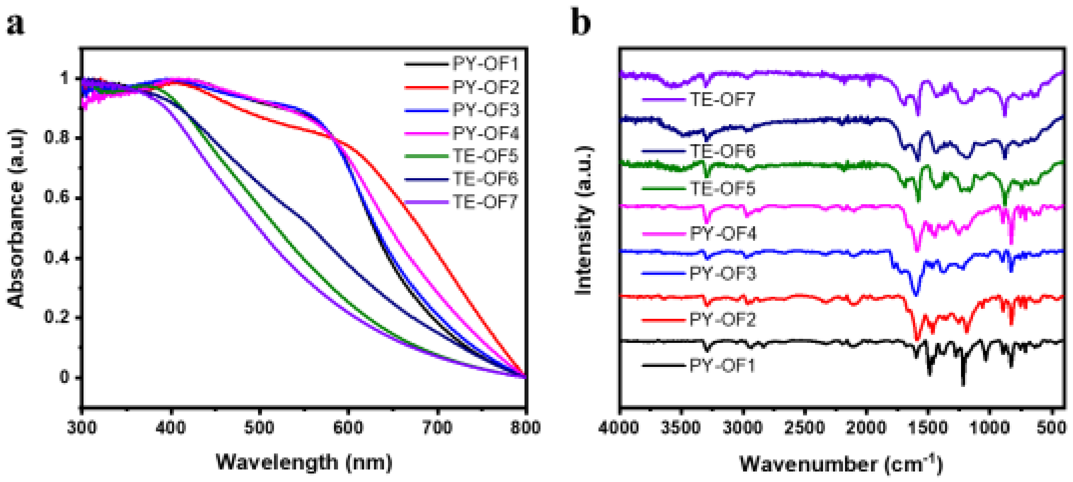

Figure S3: (a) UV-vis spectra and (b) IR spectra of PY-OF1-4 and TE-OF-5-7 CMPs.

Figure S4: UV-vis spectra and IR spectra of DE7 and DE7 derivatives

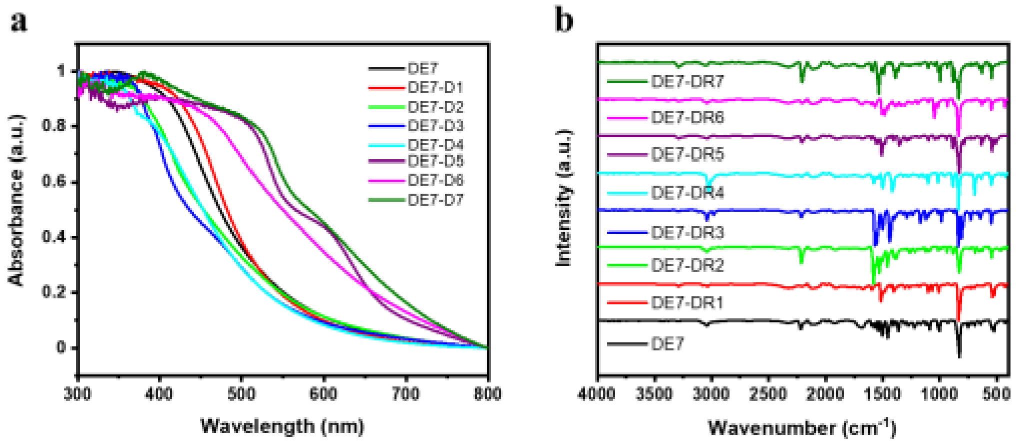

Figure S4: (a) UV-vis spectra and (b) IR spectra of DE7 and DE7 derivatives.

Figure S5: PXRD patterns and TGA data of DE7 and DE7 derivatives

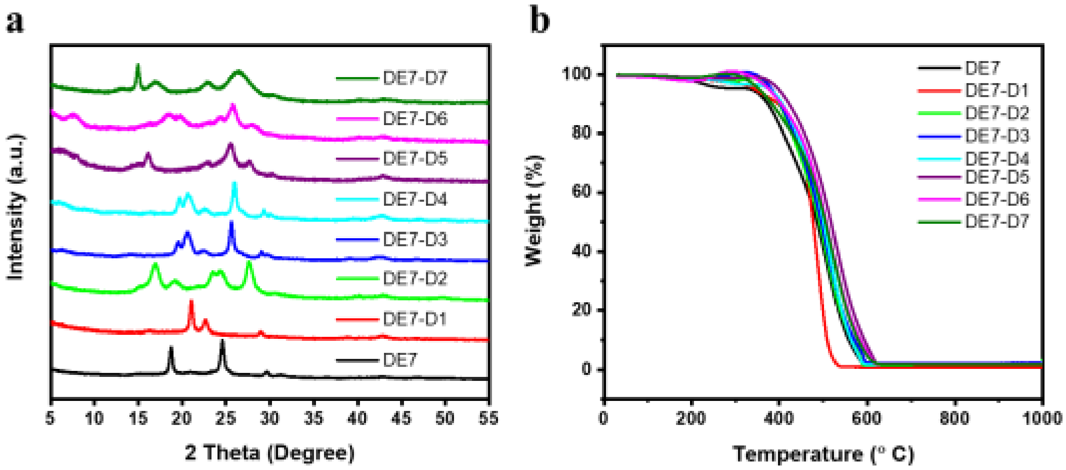

Figure S5: (a) PXRD patterns and (b) TGA data of DE7 and DE7 derivatives. All of these rigid alkynyl polymers show evidence of semicrystallinity.

Figure S6: SEM images of DE7 and DE7 derivatives

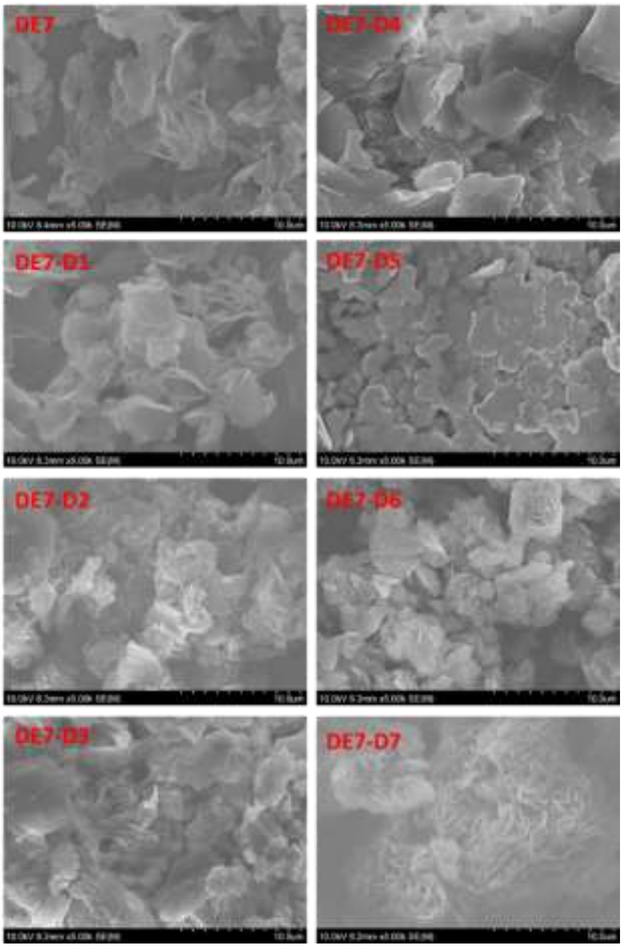

Figure S6: SEM images of DE7 and DE7 derivatives.

Figure S7: Images of contact angle against water for DE7 and DE7 derivatives

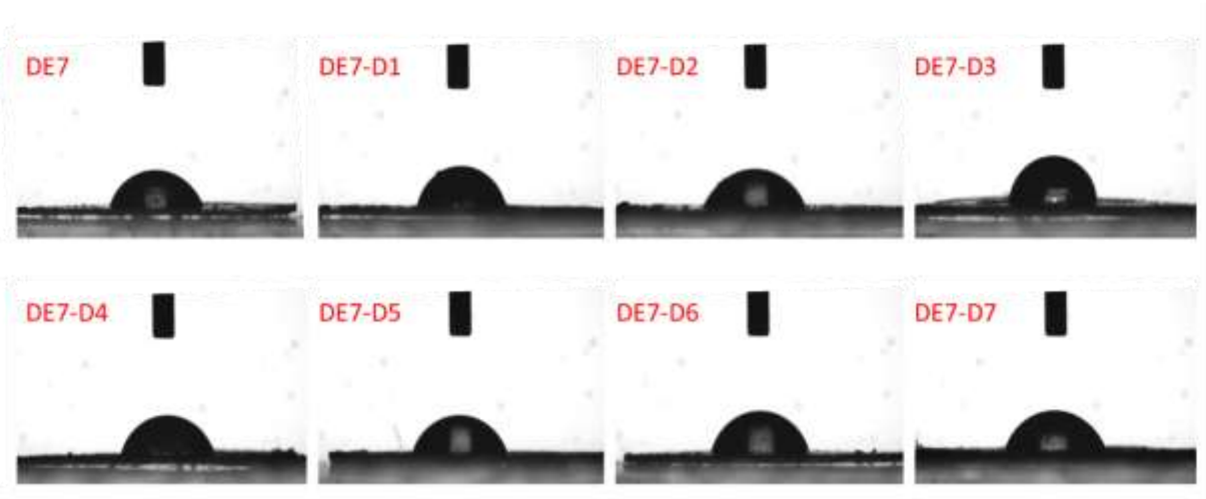

Figure S7: Contact angle against water for DE7 and DE7 derivatives.

Figure S8: Property-performance relationships for DE7 and DE7 derivatives

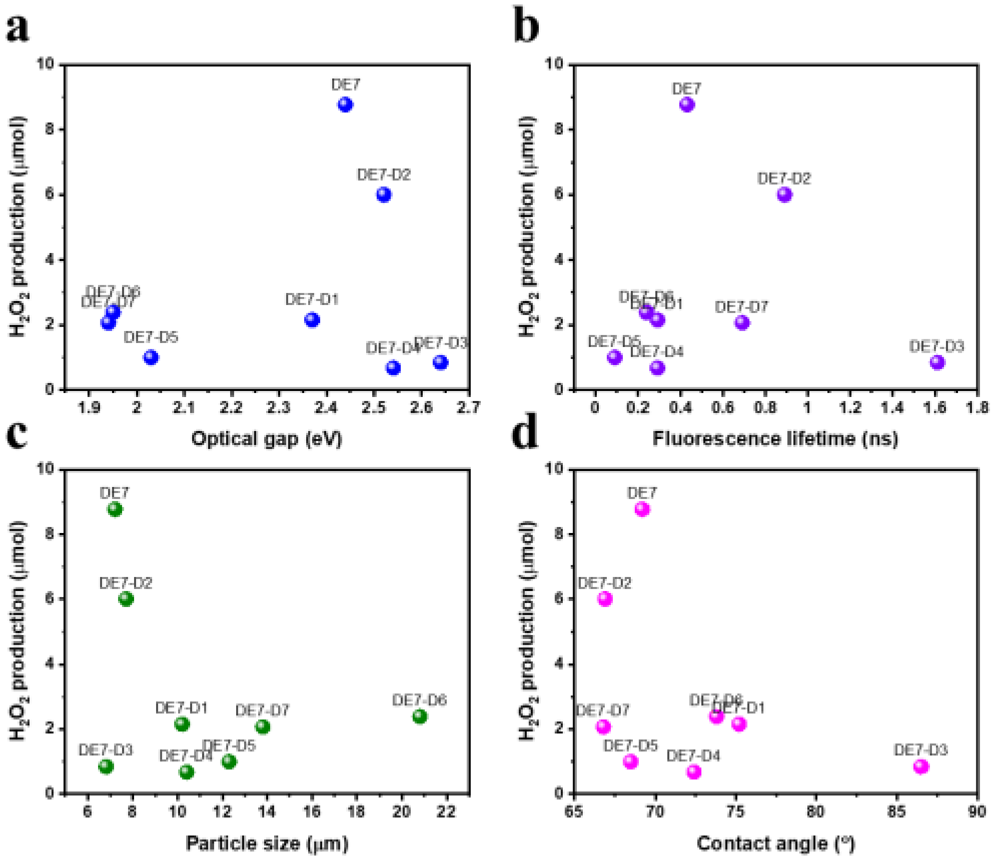

Figure S8: (a) Optical gaps, (b) fluorescence lifetimes, (c) particle size and (d) contact angle plots versus H_2O_2 production performance for DE7 and DE7 derivatives. The H_2O_2 production performance does not correlate simply with any single property.

Figure S9: Photocatalytic H_2 and H_2O_2 production of DE-n and TE-n polymers

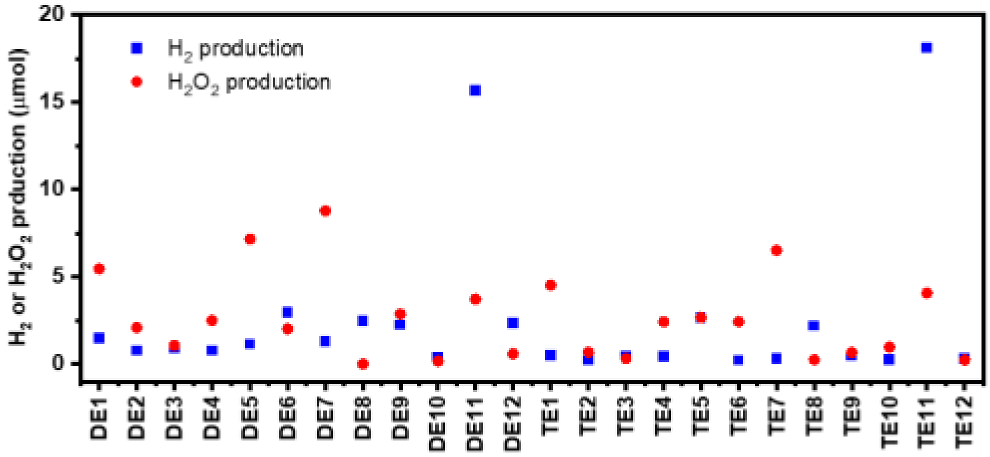

Figure S9: Photocatalytic H_2 production[5] and H_2O_2 production performance of DE 1-12 and TE1-12 polymers. H_2 production: 5 mL water/methanol/triethylamine(1:1:1) mixture solution and 5 mg polymer for 2 h illumination in N_2. H_2O_2 production: 3 mL water and 5 mg polymer for 1.5 h illumination in air. Light source: Oriel Solar Simulator 94123A with an output of 1.0 sun. The H_2O_2 production rate was measured using a KI titration method.

Figure S10: Synthesis procedures for DE7 and DE7-M

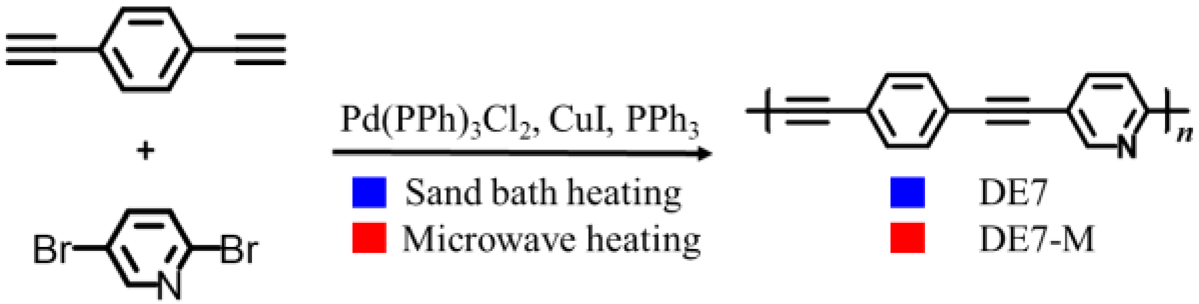

Figure S10: Synthesis procedures for DE7 and DE7-M.

Figure S11: Solid-state NMR spectra

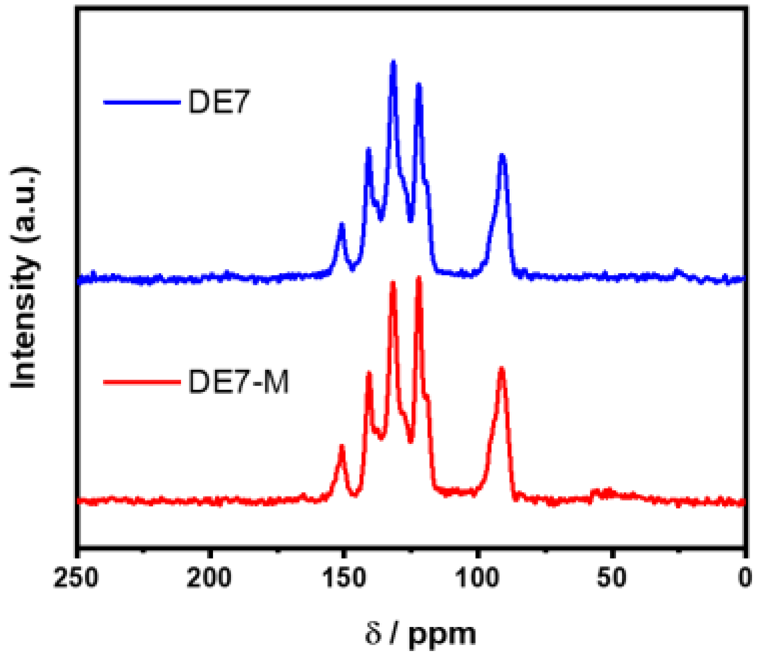

Figure S11: Solid-state NMR spectra of DE7 and DE7-M.

Figure S12: Raman spectra

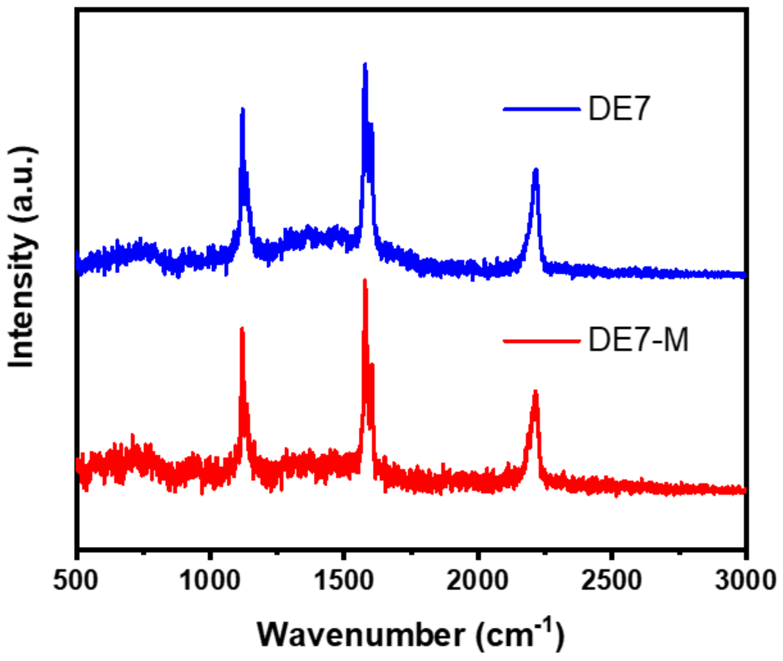

Figure S12: Raman spectra of DE7 and DE7-M.

Figure S13: XPS spectra

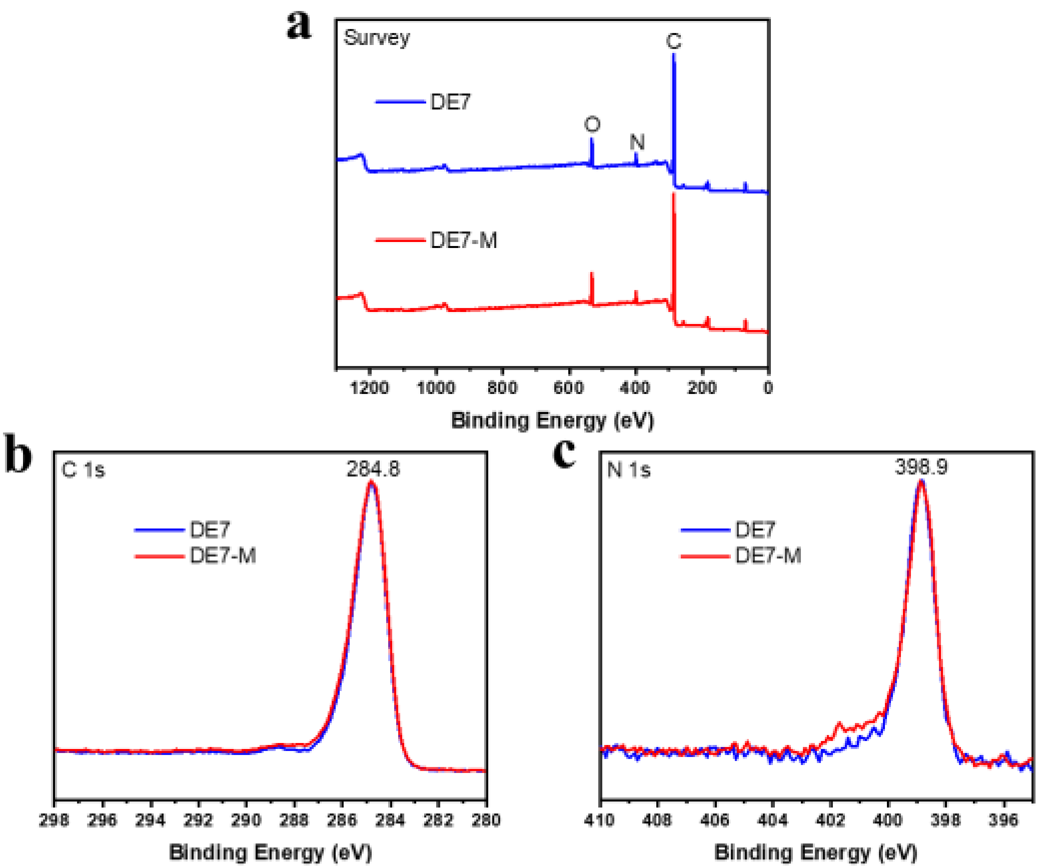

Figure S13: XPS spectra: (a) survey (b) C 1s (c) N 1s of DE7 and DE7-M.

Figure S14: Thermogravimetric data

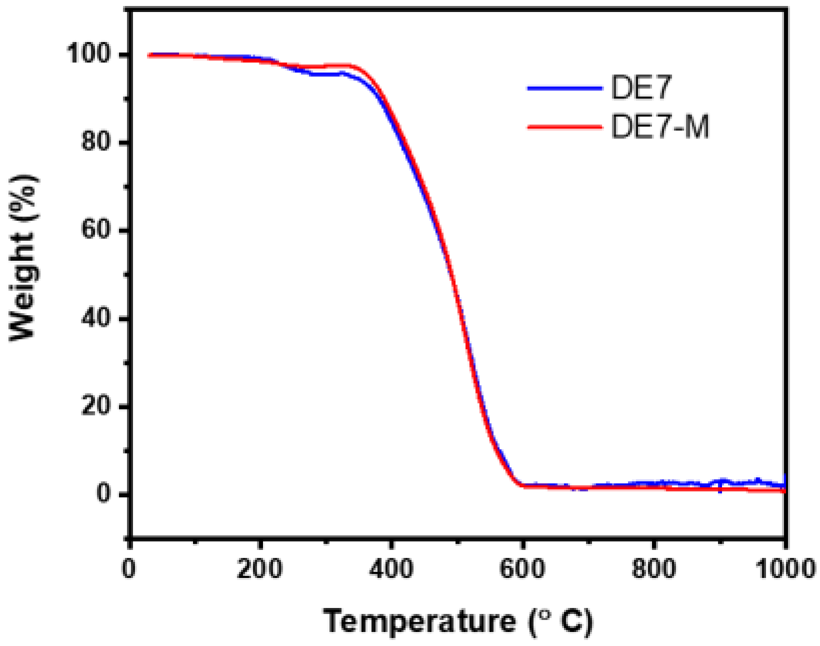

Figure S14: Thermogravimetric data in air of DE7 and DE7-M.

Figure S15: Brunauer-Emmett-Teller surface area

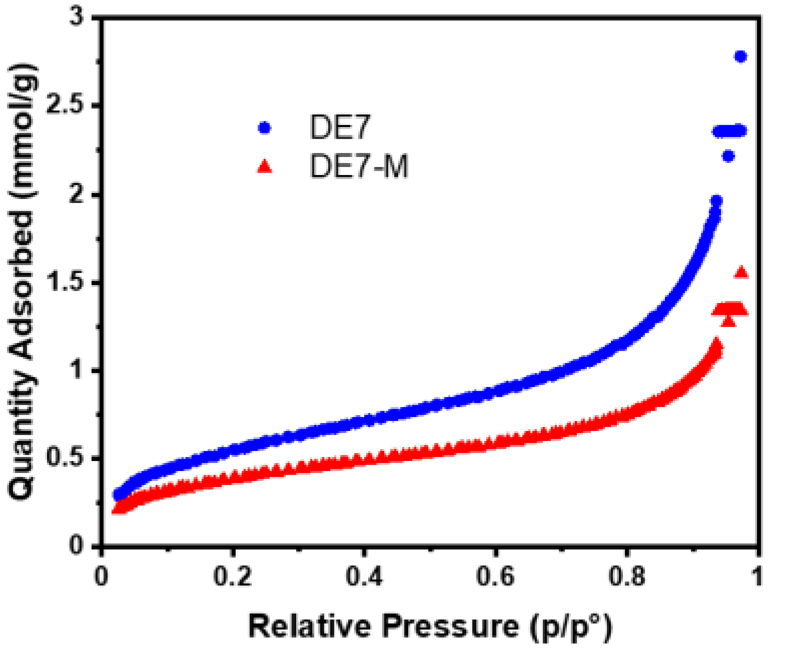

Figure S15: Nitrogen sorption isotherm of DE7 and DE7-M. They have low surface area of 46 m^2 g^{-1} (DE7) and 32 m^2 g^{-1} (DE7-M).

Figure S16: Effect of residual Pd and Cu

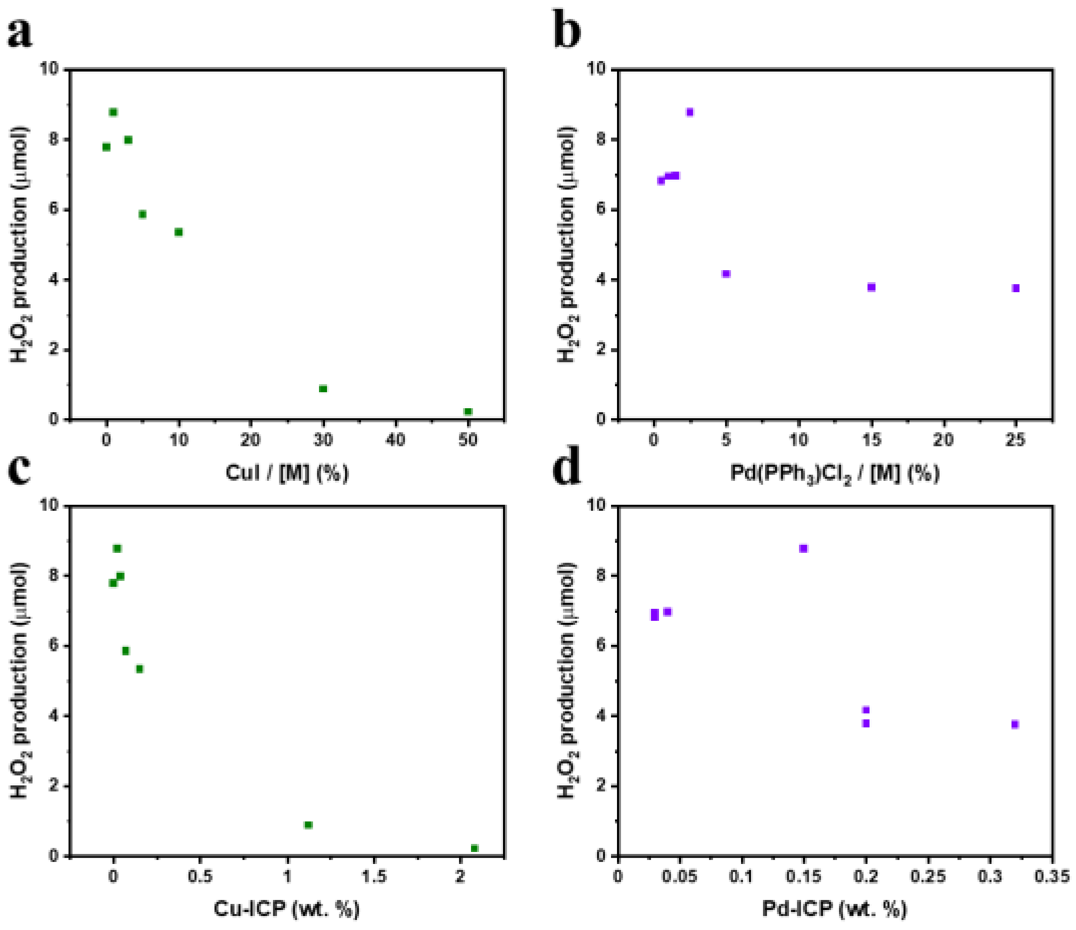

Figure S16: Photocatalytic H_2O_2 production of DE7 with different contents of (a) CuI, (b) $Pd(PPh_3)Cl_2$ and corresponding (c) Cu residues, (d) Pd residues, as detected by ICP. Reaction conditions: 3 mL water and 5 mg polymer for 1.5 h illumination, measured by KI titration method. Light source: Oriel Solar Simulator 94123A with an output of 1.0 sun.

Figure S17: Photocatalytic H_2O_2 production of DE7 and DE7-M

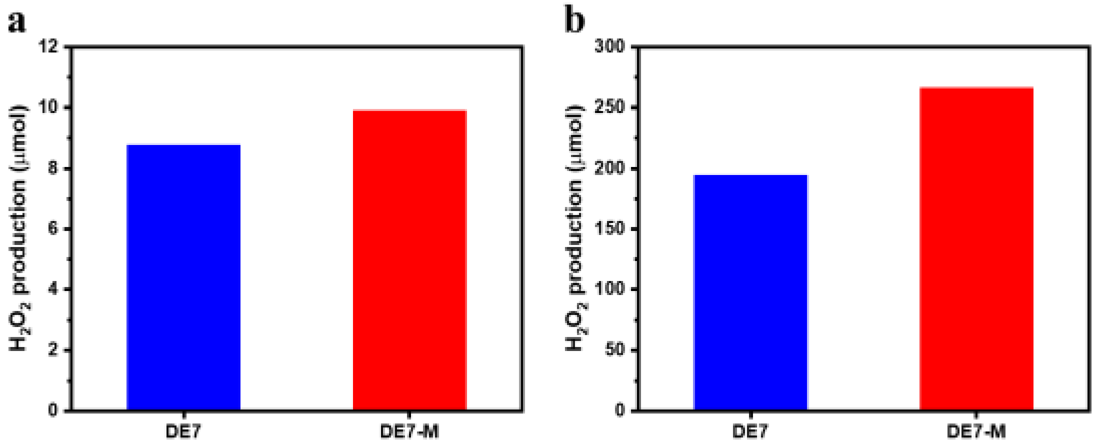

Figure S17: Photocatalytic H_2O_2 production of DE7 and DE7-M. Reaction conditions: (a) 3 mL water and 5 mg polymer for 1.5 h illumination. Light source: Oriel Solar Simulator 94123A with an output of 1.0 sun, measured by KI titration method. (b) : (b) 30 mL water and 50 mg polymer for 24 h illumination. Light source: 300 W Xe lamp with a filter (λ > 420 nm), measured by KI titration method.

Figure S18: Optoelectronic properties of DE7 and DE7-M

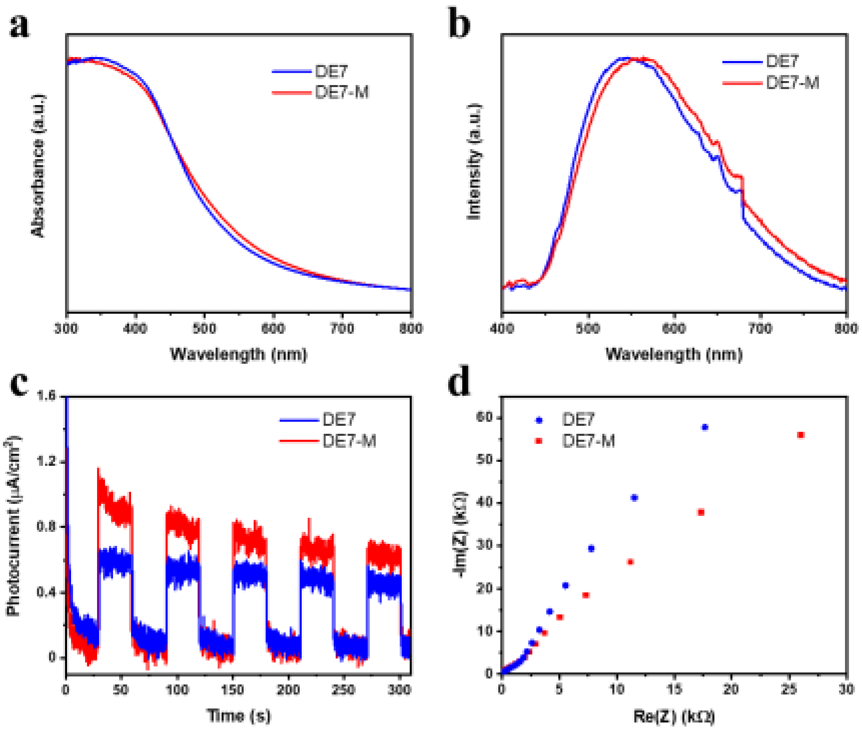

Figure S18: (a) Solid-state UV-vis spectra, (b) Photoluminescence emission spectra (λexc = 371 nm), (c) photocurrent responses and (d) electrochemical impedance spectroscopy (EIS) Nyquist plots of DE7 and DE7-M.

Figure S19: Contact angle measurements

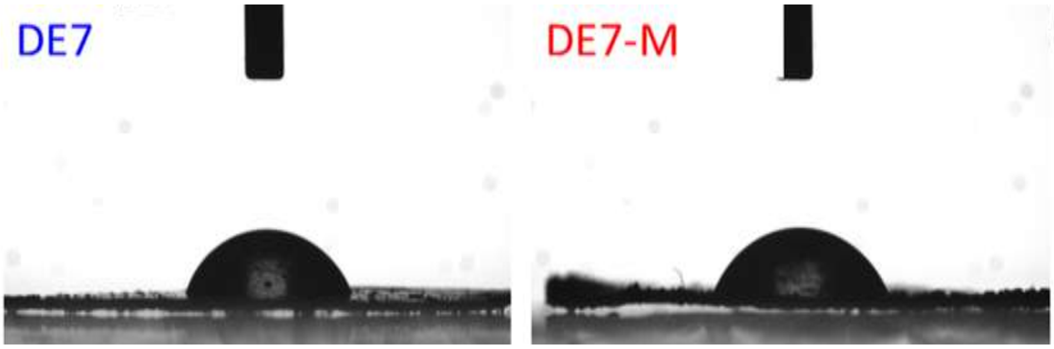

Figure S19: Contact angle against water, DE7 (left) and DE7-M (right).

Figure S20: Solar-to-chemical conversion (SCC) efficiency

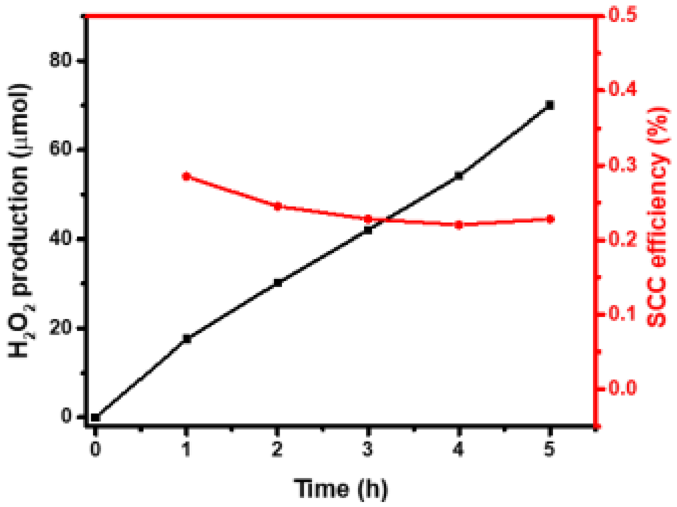

Figure S20: Photocatalytic H_2O_2 production and the SCC efficiency of DE7-M in a 40 °C water bath under a LED simulated sunlight (1 sun) irradiation. Reaction conditions: water (50 mL), catalyst (200 mg), in O_2, measured using a KI titration method.

Figure S21: Half photoreaction of DE7-M for H_2O_2 production

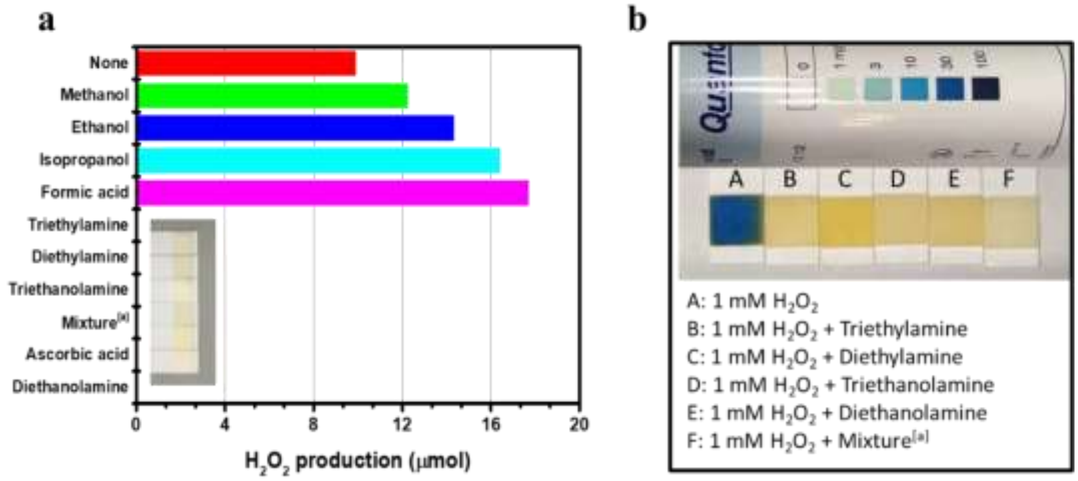

Figure S21: (a) Half reaction of DE7-M for H_2O_2 production in different electron-donor sacrificial reagents. Reaction conditions: 5 mg polymer, 2.7 mL H_2O and 0.3 mL sacrificial reagent, in air, measured by KI titration method (Inset figure in (a) shows the results confirmed by Peroxide test sticks again). Light source: Oriel Solar Simulator 94123A with an output of 1.0 sun. (b) 1 mM H_2O_2 aqueous solution (as a reference) and images for solutions after adding 10 % of various basic sacrificial reagents, as measured using Peroxide test sticks. ([a] The mixture is 1 mL triethylamine, 1 mL methanol and 1 mL water).

Figure S22: Photocatalytic H_2O_2 production of DE7-M at different pH

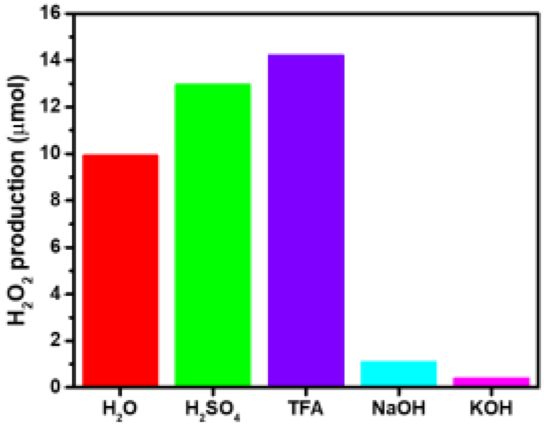

Figure S22: Photocatalytic H_2O_2 production of DE7-M under different pH conditions: DI water (H_2O, pH = 7), sulfuric acid solution (H_2SO_4, pH = 1), trifluoroacetic acid solution (TFA, pH = 1), sodium hydroxide solution (NaOH, pH = 13) and potassium hydroxide solution (KOH, pH = 14) of DE7-M. Reaction conditions: 3 mL solution and 5 mg polymer for 1.5 h illumination, measured by KI titration method. Light source: Oriel Solar Simulator 94123A with an output of 1.0 sun.

Figure S23: Photocatlaytic water oxidation half reaction for DE7-M

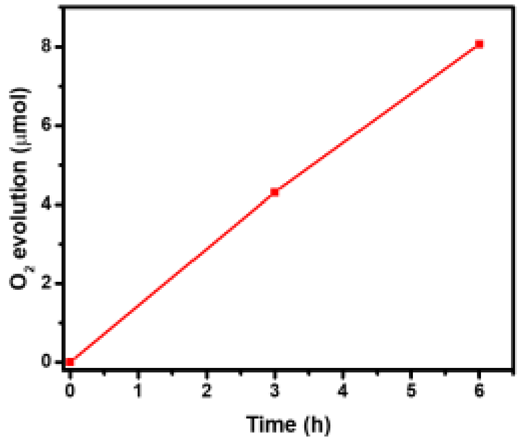

Figure S23: O_2 production in the half reaction of DE7-M. Reaction conditions: 50 mg DE7-M, 100 mL water, 10 mM $AgNO_3$ and 200 mg La_2O_3 in N_2 under visible light illumination (300 W Xe lamp with a $\lambda > 420$ nm filter).

Figure S24: Electrochemical Mott-Schottky measurements

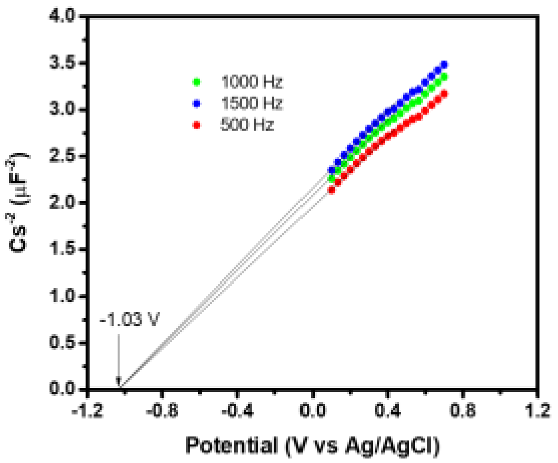

Figure S24: (a) Electrochemical Mott-Schottky plots for DE7-M. The flat band (E_{fb}) potential of DE7-M was measured by the electrochemical Mott-Schottky technique in standard three-electrode system with 0.5 M Na_2SO_4 aqueous solution (pH = 7) as the electrolyte,[6, 7] which can be further converted into -0.83 V *vs.* NHE according to the equation of $E_{NHE} = E_{Ag/AgCl} + 0.197$ V.[8, 9] In addition, the positive slope indicated that DE7-M was a n-type semiconductor. For many n-type semiconductors, E_{fb} is normally deemed to be more positive about 0.1 V than its conduction band (CB) potentials (E_{CB}).[8] Therefore, the E_{CB} of DE7-M was calculated -0.93 V *vs.* NHE. Besides, the optical gap (E_g) of DE7-M was obtained from the UV-vis spectrum with a value of 2.34 eV (2.34 V *vs.* NHE) (Figure 2c), so the valence band (VB) of DE7-M was calculated at1 1.41 V *vs.* NHE from the equation of $E_{vb} = E_{cb} + E_g$.

Figure S25: Photocatalytic decomposition of H_2O_2

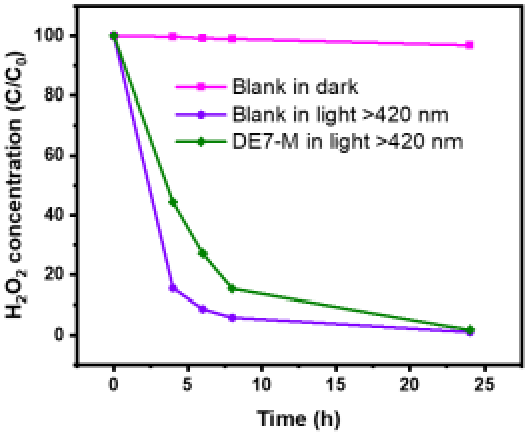

Figure S25: To check the photostability of H_2O_2, photodecomposition experiments both with and without DE7-M were carried out. Reaction conditions: 30 mL 1 mM H_2O_2 aqueous solution, without catalyst (blank) or with 50 mg DE7-M, under dark or visible light illumination (300 W Xe lamp with a $\lambda > 420$ nm filter) under a N_2 atmosphere; peroxide concentrations measured by a KI titration method. The H_2O_2 is decomposed by irradiation in the absence of oxygen, both with and without the polymer catalyst, although slightly more slowly when the catalyst is present. This explains the light-induced decomposition of H_2O_2 observed at longer photolysis times when the catalyst becomes deactivated (>50 h, Fiure 4b) or at shorter times if the reaction (and the catalyst decomposition) is accelerated using sacrificial donors (Figure S31a, below).

Figure S26: SEM images, UV-vis spectrum and FT-IR spectra for RF523

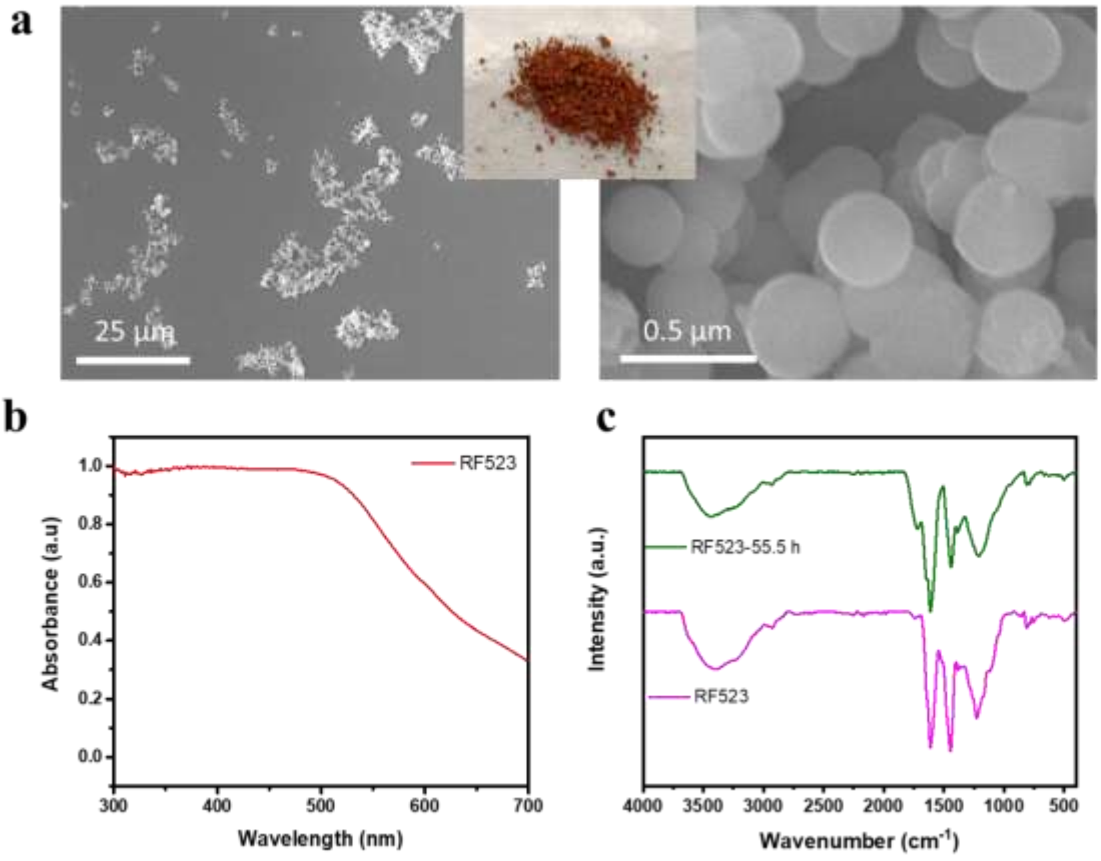

Figure S26: (a) Photograph (colored inset) and SEM images; (b) Solid UV-vis spectrum of as-synthesized RF523; (c) FT-IR spectra of as-synthesized RF523 and RF523 after 55.5 h illumination under visible light illumination (300 W Xe lamp with a $\lambda > 420$ nm filter), suggesting significant chemical changes to the polymer, which becomes almost catalytically inactive after this time (see main text, Figure 4b).

Figure S27: SEM images of DE7-M after long-term photoreaction

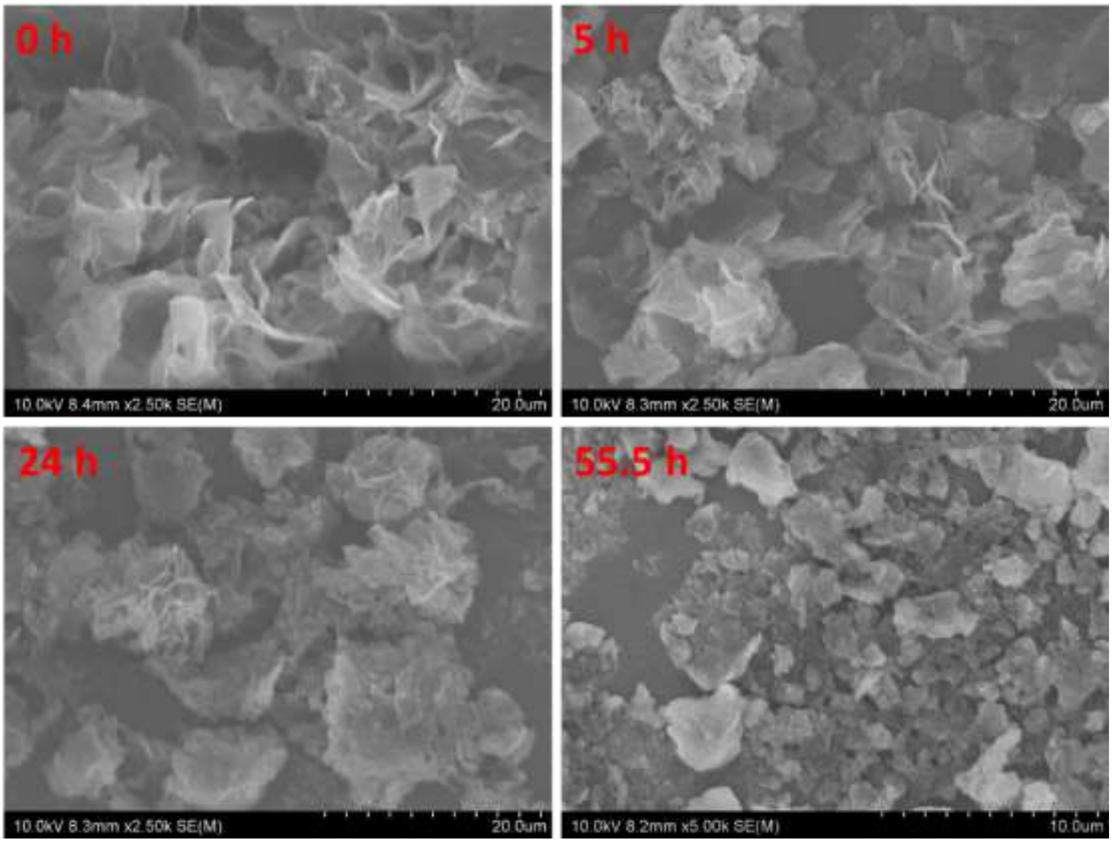

Figure S27: SEM images of fresh DE7-M and DE7-M after 5 h, 24 h, and 55.5 h illumination under visible light illumination (300 W Xe lamp with a λ > 420 nm filter).

Figure S28: PXRD patterns of DE7-M after long-term photoreaction

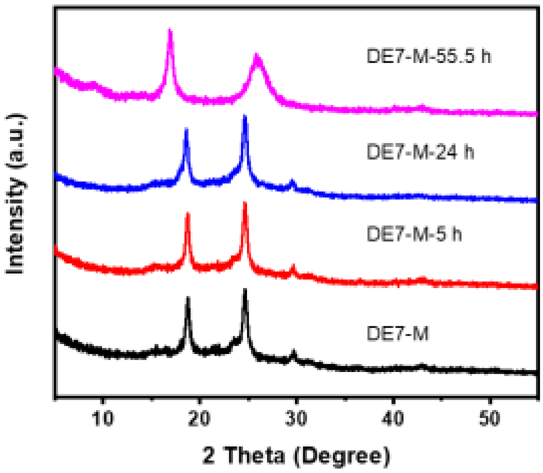

Figure S28: PXRD patterns of fresh DE7-M and DE7-M after 5 h, 24 h, and 55.5 h illumination under visible light illumination (300 W Xe lamp with a λ > 420 nm filter).

Figure S29: FT-IR spectra of DE7-M after long-term photoreaction

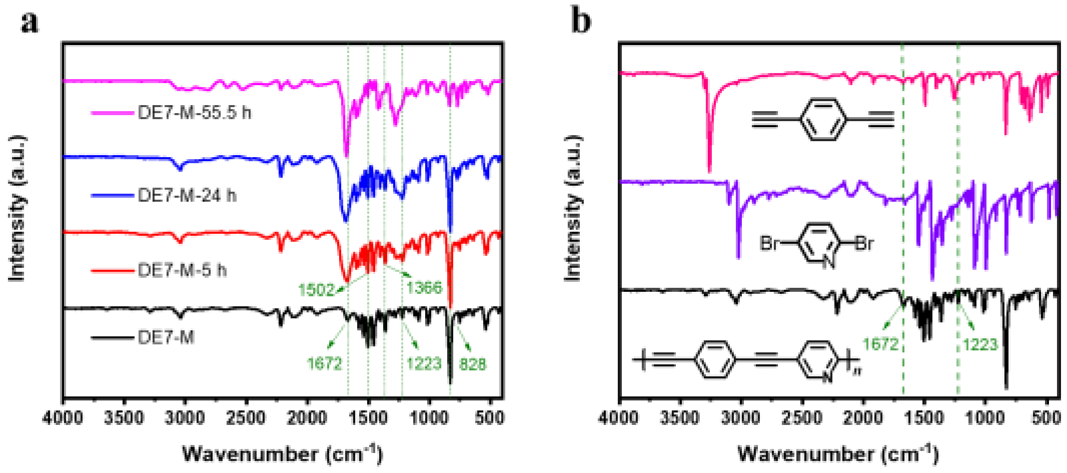

Figure S29: FT-IR spectra of (a) fresh DE7-M and DE7-M after 5 h, 24 h, and 55.5 h illumination under visible light illumination (300 W Xe lamp with a λ > 420 nm filter); (b) 2,5-dibromopyridine (99%), 1,4-diethynylbenzene (96%) and fresh DE7-M.

Figure S30: Proposed decomposition route for DE7-M

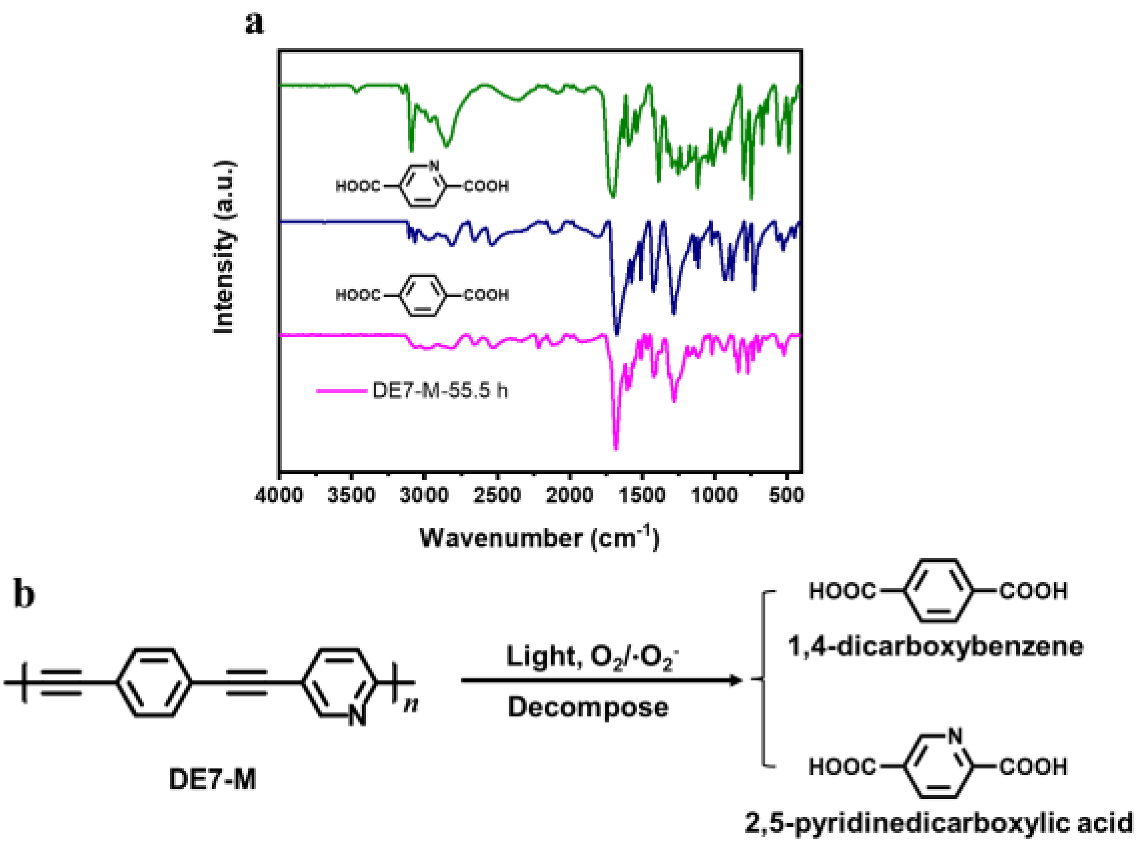

Figure S30: (a) FT-IR spectra of fresh DE7-M after 55.5 h illumination under visible light illumination and as received 1,4-dicarboxybenzene (Aldrich) and 2,5-pyridinedicarboxylic acid (Fluorochem). Reaction conditions: 50 mg polymer, 30 mL H_2O under visible light illumination (300 W Xe lamp with a $\lambda > 420$ nm filter). (b) Proposed decomposition route for DE7-M; we assume that this proceeds first via polymer cleavage and the formation of oligomers (hence the blue shift in the absorption spectrum, Figure 4c, main text); the dicarboxylic acids are the final decomposition product.[10] We note that this decomposition can also be observed by eye; there is visibly less solid catalyst material present as the reaction proceeds, as quantified by the weight loss measurements shown in Figure 4a (main text).

Figure S31: Long-term photoreaction of DE7-M with the addition of IPA

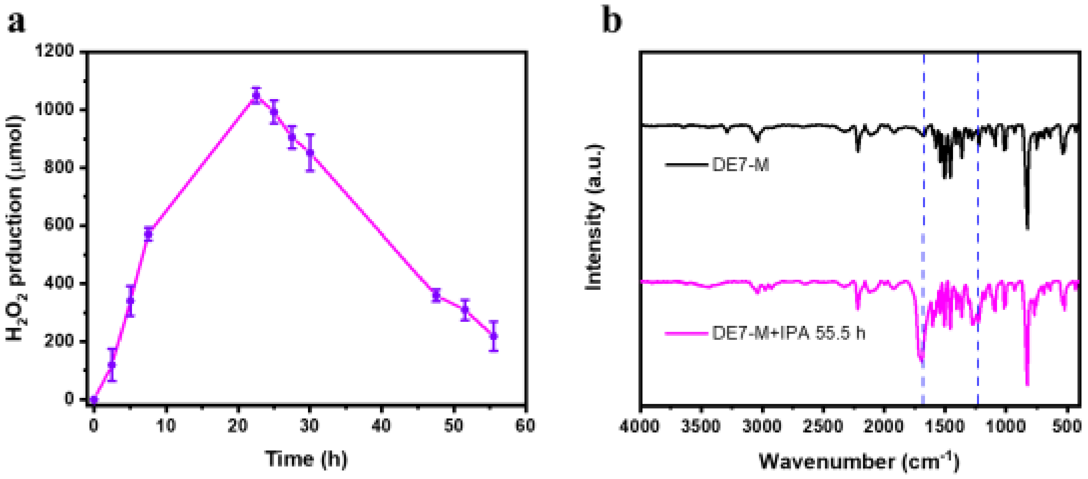

Figure S31: (a) Long-term photocatalytic H_2O_2 production of DE7-M with the addition of isopropanol (IPA). Reaction conditions: 50 mg polymer, 3 mL H_2O and 27 mL IPA under visible light illumination (300 W Xe lamp with a $\lambda > 420$ nm filter). (b) FT-IR spectra of fresh DE7-M and DE7-M after 55.5 h with the addition of IPA under visible light illumination (300 W Xe lamp with a $\lambda > 420$ nm filter). Both the photocatalytic H_2O_2 production rate and the DE7-M decomposition is accelerated by IPA. After 22.5 h, the catalyst becomes deactivated and hence the H_2O_2 is photodecomposed after that period, as in the blank experiment shown in Figure S25.

10. Supporting tables

Table S1: Cu contents of polymers synthesized via Sonogashira coupling reactions

Sample	Cu (wt.%)	Sample	Cu (wt.%)	Sample	Cu (wt.%)	Sample	Cu (wt.%)
TE1	0.04	DE1	0.01	PY-OF1	0.47	DE7-D1	0.01
TE2	0.02	DE2	0.02	PY-OF2	0.28	DE7-D2	0.01
TE3	0.004	DE3	0.04	PY-OF3	1.24	DE7-D3	0.02
TE4	0.08	DE4	0.05	PY-OF4	1.03	DE7-D4	0.03
TE5	0.19	DE5	0.09	TE-OF5	0.23	DE7-D5	0.01
TE6	0.21	DE6	0.05	TE-OF6	0.25	DE7-D6	0.03
TE7	0.13	DE7	0.02	TE-OF7	0.14	DE7-D7	0.01
TE8	0.40	DE8	0.64				
TE9	0.11	DE9	0.07				
TE10	0.04	DE10	0.06				
TE11	0.10	DE11	0.04				
TE12	0.20	DE12	0.29				

Table S2: Comparation of the catalytic H_2O_2 production activity of DE7-M with other reported organic materials[a]

No.	Material	H_2O_2 yield / μmol	SCC / %	AQY / %[b]	Reaction conditions	Ref.
1	DE7-M	266 (24 h)	0.23	8.7	λ > 420 nm	This work
2	RF523	61.6 (24 h)	0.5	~8.0	λ > 420 nm	3
3	RF-acid resins	91.0 (24 h)	0.7	8.5	λ > 420 nm	11
4	RF/P3HT resin	100 (18 h)	1.0	10.5	λ > 420 nm	12
5	CTF-BDDBN	70 (24 h)	0.14	–	λ > 420 nm	13
6	OCN-500	53 (10 h)	–	10.2	λ > 420 nm	4
7	Sb-SAPC15	470.5 (8 h)	0.61	17.6	λ > 420 nm	14
8	g-C_3N_4/PDI/$rGO_{0.05}$	29 (24 h)	0.20	6.1	λ > 420 nm	15
9	PEI/C_3N_4	4.2 (1 h)	0.045	2.21	λ > 420 nm	16
10	R_{370}-CN	17 (1 h)	~0.26	~4.3	λ > 420 nm	17
11	PCNBA0.2Co5%	~35 (12 h)	0.30	8.0	λ > 420 nm	18

[a] The detailed measurement conditions for H_2O_2 yield, SCC and AQY vary in different reports, and the nature and intensity of the light source, for example, may be very important. Also, few studies show experiments that last for more than 24 h, and many focus on the first 1-12 h; efficiencies might be much lower over longer timescales. Experiments that show "recycling" tests, where the aqueous solution is periodically replaced after short intervals (1-2 h), might in fact mask catalyst instability.[3, 11, 12, 16, 19, 20, 21, 22, 23, 24, 25] They do not in any case demonstrate "recycling" because these time intervals are far too short.

[b] All reported at 420 nm.

Table S3: Elemental analysis results of DE7-M after long-term photoreaction

DE7-M[a]	%C	%H	%N
0 h	74.98	3.50	6.85
5 h	72.37	3.37	6.27
24 h	69.12	3.07	5.77
55.5 h	68.36	3.05	5.56

[a] Fresh DE7-M (0 h) and DE7-M after 5 h, 24 h, and 55.5 h illumination under a Xe lamp illumination with a filter ($\lambda > 420$ nm).

11. References

(1) Wang, P.; Xu, Q.; Li, Z.; Jiang, W.; Jiang, Q.; Jiang, D., Exceptional Iodine Capture in 2D Covalent Organic Frameworks. *Adv. Mater.* **2018,** *30* (29), 1801991.
(2) Liu, L.; Kochman, M. A.; Xu, Y.; Zwijnenburg, M. A.; Cooper, A. I.; Sprick, R. S., Acetylene-linked conjugated polymers for sacrificial photocatalytic hydrogen evolution from water. *J. Mater. Chem. A* **2021,** *9* (32), 17242-17248.
(3) Shiraishi, Y.; Takii, T.; Hagi, T.; Mori, S.; Kofuji, Y.; Kitagawa, Y.; Tanaka, S.; Ichikawa, S.; Hirai, T., Resorcinol–formaldehyde resins as metal-free semiconductor photocatalysts for solar-to-hydrogen peroxide energy conversion. *Nat. Mater.* **2019,** *18* (9), 985-993.
(4) Wei, Z.; Liu, M.; Zhang, Z.; Yao, W.; Tan, H.; Zhu, Y., Efficient visible-light-driven selective oxygen reduction to hydrogen peroxide by oxygen-enriched graphitic carbon nitride polymers. *Energy Environ. Sci.* **2018,** *11* (9), 2581-2589.
(5) Liu, L.; Kochman, M. A.; Xu, Y.; Zwijnenburg, M. A.; Cooper, A. I.; Sprick, R. S., Acetylene-linked conjugated polymers for sacrificial photocatalytic hydrogen evolution from water. *J. Mater. Chem. A* **2021**.
(6) Zhang, G.; Lan, Z.-A.; Lin, L.; Lin, S.; Wang, X., Overall water splitting by Pt/g-C3N4 photocatalysts without using sacrificial agents. *Chem. Sci.* **2016,** *7* (5), 3062-3066.
(7) Gelderman, K.; Lee, L.; Donne, S. W., Flat-Band Potential of a Semiconductor: Using the Mott–Schottky Equation. *J. Chem. Educ.* **2007,** *84* (4), 685.
(8) Wan, C.; Zhou, L.; Sun, L.; Xu, L.; Cheng, D.-g.; Chen, F.; Zhan, X.; Yang, Y., Boosting visible-light-driven hydrogen evolution from formic acid over AgPd/2D g-C3N4 nanosheets Mott-Schottky photocatalyst. *Chem. Eng. J.* **2020,** *396*, 125229.
(9) Zhang, M.; Lu, M.; Lang, Z.-L.; Liu, J.; Liu, M.; Chang, J.-N.; Li, L.-Y.; Shang, L.-J.; Wang, M.; Li, S.-L.; Lan, Y.-Q., Semiconductor/Covalent-Organic-Framework Z-Scheme Heterojunctions for Artificial Photosynthesis. *Angew. Chem. Int. Ed.* **2020,** *59* (16), 6500-6506.
(10) Tian, S.; Yue, Q.; Liu, C.; Li, M.; Yin, M.; Gao, Y.; Meng, F.; Tang, B. Z.; Luo, L., Complete Degradation of a Conjugated Polymer into Green Upcycling Products by Sunlight in Air. *J. Am. Chem. Soc.* **2021,** *143* (27), 10054-10058.
(11) Shiraishi, Y.; Hagi, T.; Matsumoto, M.; Tanaka, S.; Ichikawa, S.; Hirai, T., Solar-to-hydrogen peroxide energy conversion on resorcinol–formaldehyde resin photocatalysts prepared by acid-catalysed polycondensation. *Commun. Chem.* **2020,** *3* (1), 169.
(12) Shiraishi, Y.; Matsumoto, M.; Ichikawa, S.; Tanaka, S.; Hirai, T., Polythiophene-Doped Resorcinol–Formaldehyde Resin Photocatalysts for Solar-to-Hydrogen Peroxide Energy Conversion. *J. Am. Chem. Soc.* **2021,** *143* (32), 12590-12599.
(13) Chen, L.; Wang, L.; Wan, Y.; Zhang, Y.; Qi, Z.; Wu, X.; Xu, H., Acetylene and Diacetylene Functionalized Covalent Triazine Frameworks as Metal-Free Photocatalysts for Hydrogen Peroxide Production: A New Two-Electron Water Oxidation Pathway. *Adv. Mater.* **2020,** *32* (2), 1904433.
(14) Teng, Z.; Zhang, Q.; Yang, H.; Kato, K.; Yang, W.; Lu, Y.-R.; Liu, S.; Wang, C.; Yamakata, A.; Su, C.; Liu, B.; Ohno, T., Atomically dispersed antimony on carbon nitride for the artificial photosynthesis of hydrogen peroxide. *Nat. Catal.* **2021,** *4* (5), 374-384.
(15) Kofuji, Y.; Isobe, Y.; Shiraishi, Y.; Sakamoto, H.; Tanaka, S.; Ichikawa, S.; Hirai, T., Carbon Nitride–Aromatic Diimide–Graphene Nanohybrids: Metal-Free Photocatalysts for Solar-to-Hydrogen Peroxide Energy Conversion with 0.2% Efficiency. *J. Am. Chem. Soc.* **2016,** *138* (31), 10019-10025.
(16) Zeng, X.; Liu, Y.; Kang, Y.; Li, Q.; Xia, Y.; Zhu, Y.; Hou, H.; Uddin, M. H.; Gengenbach, T. R.; Xia, D.; Sun, C.; McCarthy, D. T.; Deletic, A.; Yu, J.; Zhang, X., Simultaneously Tuning Charge Separation and Oxygen Reduction Pathway on Graphitic Carbon Nitride by

Polyethylenimine for Boosted Photocatalytic Hydrogen Peroxide Production. *ACS Catal.* **2020,** *10* (6), 3697-3706.
(17) Zhu, Z.; Pan, H.; Murugananthan, M.; Gong, J.; Zhang, Y., Visible light-driven photocatalytically active g-C3N4 material for enhanced generation of H2O2. *Appl. Catal. B-Environ.* **2018,** *232*, 19-25.
(18) Teng, Z.; Cai, W.; Liu, S.; Wang, C.; Zhang, Q.; Chenliang, S.; Ohno, T., Bandgap engineering of polymetric carbon nitride copolymerized by 2,5,8-triamino-tri-s-triazine (melem) and barbituric acid for efficient nonsacrificial photocatalytic H2O2 production. *Appl. Catal. B-Environ.* **2020,** *271*, 118917.
(19) Kim, H.-i.; Choi, Y.; Hu, S.; Choi, W.; Kim, J.-H., Photocatalytic hydrogen peroxide production by anthraquinone-augmented polymeric carbon nitride. *Appl. Catal. B-Environ.* **2018,** *229*, 121-129.
(20) Liu, W.; Song, C.; Kou, M.; Wang, Y.; Deng, Y.; Shimada, T.; Ye, L., Fabrication of ultra-thin g-C3N4 nanoplates for efficient visible-light photocatalytic H2O2 production via two-electron oxygen reduction. *Chem. Eng. J.* **2021,** *425*, 130615.
(21) Wu, S.; Yu, H.; Chen, S.; Quan, X., Enhanced Photocatalytic H2O2 Production over Carbon Nitride by Doping and Defect Engineering. *ACS Catal.* **2020,** *10* (24), 14380-14389.
(22) Gryszel, M.; Sytnyk, M.; Jakešová, M.; Romanazzi, G.; Gabrielsson, R.; Heiss, W.; Głowacki, E. D., General Observation of Photocatalytic Oxygen Reduction to Hydrogen Peroxide by Organic Semiconductor Thin Films and Colloidal Crystals. *ACS Appl. Mater. Interfaces* **2018,** *10* (16), 13253-13257.
(23) Luo, J.; Liu, Y.; Fan, C.; Tang, L.; Yang, S.; Liu, M.; Wang, M.; Feng, C.; Ouyang, X.; Wang, L.; Xu, L.; Wang, J.; Yan, M., Direct Attack and Indirect Transfer Mechanisms Dominated by Reactive Oxygen Species for Photocatalytic H2O2 Production on g-C3N4 Possessing Nitrogen Vacancies. *ACS Catal.* **2021**, 11440-11450.
(24) Lin, S.; Zhang, N.; Wang, F.; Lei, J.; Zhou, L.; Liu, Y.; Zhang, J., Carbon Vacancy Mediated Incorporation of Ti3C2 Quantum Dots in a 3D Inverse Opal g-C3N4 Schottky Junction Catalyst for Photocatalytic H2O2 Production. *ACS Sustainable Chem. Eng.* **2021,** *9* (1), 481-488.
(25) Zheng, L.; Zhang, J.; Hu, Y. H.; Long, M., Enhanced Photocatalytic Production of H2O2 by Nafion Coatings on S,N-Codoped Graphene-Quantum-Dots-Modified TiO2. *J. Phys. Chem. C* **2019,** *123* (22), 13693-13701.
